# A gain-of-function mutation in zinc cluster transcription factor Rob1 drives *Candida albicans* adaptive growth in the cystic fibrosis lung environment

**DOI:** 10.1371/journal.ppat.1012154

**Published:** 2024-04-11

**Authors:** Mayssa Gnaien, Corinne Maufrais, Yasmine Rebai, Aicha Kallel, Laurence Ma, Samia Hamouda, Fatma Khalsi, Khaoula Meftah, Hanen Smaoui, Monia Khemiri, Sondes Hadj Fredj, Sophie Bachellier-Bassi, Imène Najjar, Taieb Messaoud, Khadija Boussetta, Kalthoum Kallel, Helmi Mardassi, Christophe d’Enfert, Marie-Elisabeth Bougnoux, Sadri Znaidi

**Affiliations:** 1 Institut Pasteur de Tunis, University of Tunis El Manar, Laboratoire de Microbiologie Moléculaire, Vaccinologie et Développement Biotechnologique (LR16IPT01), Tunis, Tunisia; 2 Institut Pasteur, Université Paris Cité, INRAE USC2019A, Département Mycologie, Unité Biologie et Pathogénicité Fongiques, Paris, France; 3 Institut Pasteur, Université Paris Cité, Bioinformatics and Biostatistics Hub, F-75015 Paris, France; 4 Hôpital La Rabta, Laboratoire de Parasitologie et de Mycologie, UR17SP03, Tunis, Tunisia; 5 Institut Pasteur, Université Paris Cité, Biomics core facility, Centre de Ressources et Recherche Technologique (C2RT), Paris, France; 6 Hôpital d’Enfants Béchir Hamza de Tunis, Tunis, Tunisia; University of Würzburg, GERMANY

## Abstract

*Candida albicans* chronically colonizes the respiratory tract of patients with Cystic Fibrosis (CF). It competes with CF-associated pathogens (*e*.*g*. *Pseudomonas aeruginosa*) and contributes to disease severity. We hypothesize that *C*. *albicans* undergoes specific adaptation mechanisms that explain its persistence in the CF lung environment. To identify the underlying genetic and phenotypic determinants, we serially recovered 146 *C*. *albicans* clinical isolates over a period of 30 months from the sputum of 25 antifungal-naive CF patients. Multilocus sequence typing analyses revealed that most patients were individually colonized with genetically close strains, facilitating comparative analyses between serial isolates. We strikingly observed differential ability to filament and form monospecies and dual-species biofilms with *P*. *aeruginosa* among 18 serial isolates sharing the same diploid sequence type, recovered within one year from a pediatric patient. Whole genome sequencing revealed that their genomes were highly heterozygous and similar to each other, displaying a highly clonal subpopulation structure. Data mining identified 34 non-synonymous heterozygous SNPs in 19 open reading frames differentiating the hyperfilamentous and strong biofilm-former strains from the remaining isolates. Among these, we detected a glycine-to-glutamate substitution at position 299 (G299E) in the deduced amino acid sequence of the zinc cluster transcription factor *ROB1* (*ROB1*^G299E^), encoding a major regulator of filamentous growth and biofilm formation. Introduction of the G299E heterozygous mutation in a co-isolated weak biofilm-former CF strain was sufficient to confer hyperfilamentous growth, increased expression of hyphal-specific genes, increased monospecies biofilm formation and increased survival in dual-species biofilms formed with *P*. *aeruginosa*, indicating that *ROB1*^G299E^ is a gain-of-function mutation. Disruption of *ROB1* in a hyperfilamentous isolate carrying the *ROB1*^G299E^ allele abolished hyperfilamentation and biofilm formation. Our study links a single heterozygous mutation to the ability of *C*. *albicans* to better survive during the interaction with other CF-associated microbes and illuminates how adaptive traits emerge in microbial pathogens to persistently colonize and/or infect the CF-patient airways.

## Author summary

Cystic fibrosis (CF) patients are chronically colonized with microbial species that contribute to disease severity (*e*.*g*. *Pseudomonas aeruginosa*) and engage in polymicrobial interactions to survive and better compete within the CF lung environment. *Candida albicans* is one of the CF-associated fungal species that also persists within the CF airways and is co-isolated with *P*. *aeruginosa* from CF patients. We hypothesize that the CF polymicrobial environment exerts a selective pressure on colonizing *C*. *albicans* cells, leading to the emergence of adapted strains with improved capabilities in interacting with CF-associated microbes and increased survival. Our analysis of a panel of serial *C*. *albicans* isolates from a pediatric CF patient allowed us to identify a subset of genetically closely-related strains with an enhanced ability to form both monospecies and dual-species biofilms with *P*. *aeruginosa*. We demonstrate that this trait is caused by the acquisition of a heterozygous mutation in a transcription factor-encoding gene named *ROB1* that regulates filamentous growth and biofilm development in *C*. *albicans*. Therefore, our study links a single heterozygous mutation to the emergence of an adaptive trait in *C*. *albicans* and underscores the importance of polymicrobial interactions in shaping the evolutionary trajectory of microbial pathogens while colonizing the CF airways.

## Introduction

Cystic Fibrosis (CF) is a genetic destructive multisystem disease particularly affecting the lungs and digestive tract, due to mutations altering the function of the Cystic Fibrosis Transmembrane Conductance Regulator [1]. CF patients experience thick mucus accumulation in their airways, which makes them highly susceptible to recurrent respiratory tract infections and respiratory failure. Until recently, researchers and clinicians have mainly focused on CF-associated infections caused by bacterial species, such as *Pseudomonas aeruginosa* and *Staphylococcus aureus* [2]. However, an increasing number of studies point to a clear role of fungal microorganisms, particularly *Aspergillus fumigatus* and *Candida albicans*, in the progression of the disease and its prognosis [3]. *P*. *aeruginosa* and *S*. *aureus* coexist with *Candida* spp. more frequently in sputum samples of CF patients compared with patients with other respiratory disorders [4], making *Candida* species potential players in CF polymicrobial interactions. Consistently, recent key findings indicate that CF patients with chronic *C*. *albicans* airway colonization develop a more severe lung disease [5,6] and display increased chitinase (chitotriosidase) activity associated with *C*. *albicans* in their sera and airway fluids [7], suggesting the presence of protective mechanisms in CF patients that are directed against *C*. *albicans*. Yet, CF-patient neutrophils appear to be less efficient at clearing *C*. *albicans* colonization, displaying dysfunctional swarming [8]–a mechanism employed by neutrophils to attack fungi that are larger than their size [9]. Moreover, chronic *C*. *albicans* colonization is not affected by antibiotic use or clinical exacerbation [10,11], suggesting the participation of *C*. *albicans* endogenous factors or polymicrobial interactions.

Additional studies revealed the selection of adaptation mechanisms in *C*. *albicans* clinical isolates to specifically express pathogenicity-associated traits during the course of CF disease progression, such as the acquisition of mutations conferring constitutive filamentous growth, allowing *C*. *albicans* to efficiently compete with *P*. *aeruginosa* in the CF patient airways [10] and the induction of *P*. *aeruginosa* biofilm formation on airway epithelial cells that are co-colonized by *C*. *albicans* and *P*. *aeruginosa* [12]. While the competition between *C*. *albicans* and *P*. *aeruginosa* within mixed biofilms can trigger virulence and mutability in both species [13], *C*. *albicans* was shown to enhance antibiotic tolerance of *P*. *aeruginosa* in dual-species biofilms [14]—a mechanism apparently mediated by the *C*. *albicans* cell wall polysaccharides and glycoproteins [14]. This further reflects the crucial role of polymicrobial interactions in CF-associated infections.

Although such investigations reinforce the clinical impact of *C*. *albicans* on CF pulmonary disease severity and progression, and suggest that *C*. *albicans* engages in intimate and dynamic interactions with CF-associated pathogens, the underlying molecular and phenotypic determinants are still far from being totally elucidated. We propose that *C*. *albicans* undergoes a selective pressure in the CF lung, leading to the emergence of CF-adapted *C*. *albicans* strains with altered genotypes and phenotypes. We hypothesize that chronic colonization of the CF-patient airways by *C*. *albicans* could be facilitated by specific adaptation mechanisms, involving the selection of mutations and/or activation of particular molecular pathways that could mediate the interaction of *C*. *albicans* with other microbial species colonizing the CF lung, including *P*. *aeruginosa*. In this report, we provide evidence supporting this hypothesis.

## Results

### Population structure analysis of *C*. *albicans* CF clinical isolates

We recruited 25 antifungal-naive CF patients, 2 adults and 23 pediatric patients (age range 2–22 years), over a period of ~2.5 years (Dec. 2016 to May 2019) from the Béchir Hamza Children’s Hospital in Tunis (BHCHT), a referal center for CF-patient care and treatment in Tunisia. Sputum samples were serially collected as part of the regular clinical examinations performed at the BHCHT and were directly used for the selective isolation of yeast species (S1 Table, see Materials and Methods). In total, 146 *C*. *albicans* clinical isolates were recovered from 15 patients out of 25 (S1 Table). Using previously established criteria for chronic colonization by *C*. *albicans* [6] (see Materials and Methods), 7 patients were found to chronically carry *C*. *albicans* in their airways (patients CF02, CF03, CF04, CF05, CF06, CF07 and CF12, S1 Table).

**Fig 1:**
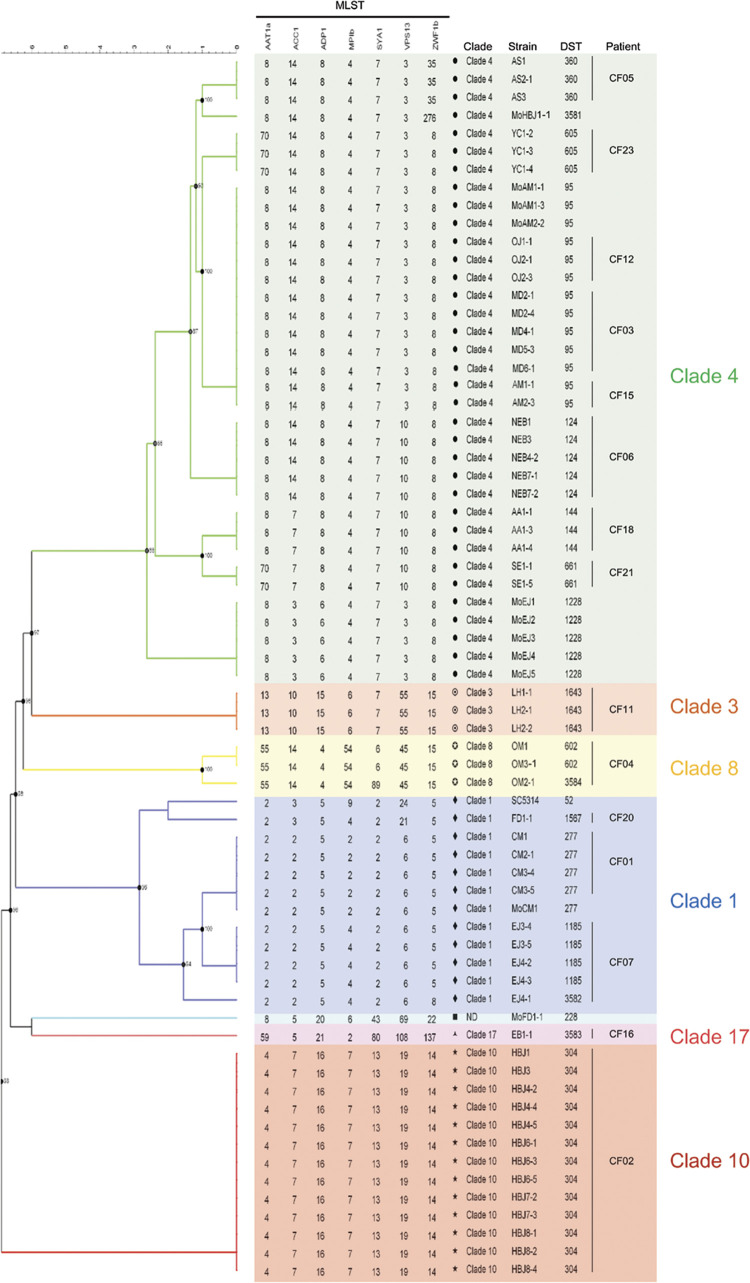
Dendrogram of the multilocus sequence types of *C*. *albicans* strains isolated from the airways of patients with CF. Phylogenetic relationship of 56 *C*. *albicans* clinical isolates recovered from the airways of 15 CF patients (CF01, CF02, CF03, CF04, CF05, CF06, CF07, CF11, CF12, CF15, CF16, CF18, CF20, CF21 and CF23) together with 11 isolates from the mothers of (Mo) patients CF01, CF02, CF07, CF15 and CF20 and reference strain SC5314. Allelic concatenated nucleic acid sequences of the seven loci (MLST, *AAT1a*, *ACC1*, *ADP1*, *MPIb*, *SYA1*, *VPS13*, and *ZWF1b*) in each strain were phylogenetically analyzed by the Bionumerics 6.0 algorithm (Applied Maths NV, St. Martens-Latem), using the categorical similarity coefficient and the UPGMA clustering method. The allelic profiles were composed of an allele identification number for each gene. An allele combination, known as diploid sequence type (DST), was assigned for the seven loci of each isolate (or obtained for isolates with new DST numbers: MoHBJ, 3581; EJ4-1, 3582; EB1-1, 3583; OM2-1, 3584) according to the *C*. *albicans* MLST database. Clade numbers were determined according to the goeBUST algorithm. Seven clades were identified, including clade 4 (green), clade 1 (blue), clade 8 (yellow), clade 3 (orange), clade 17 (pink) and clade 10 (red). Isolate MoFD1-1 clade was not determined, ND (light blue, DST228).

To better understand the epidemiology and population structure of the CF isolates, we performed multilocus sequence typing (MLST) of 56 strains originating from the 15 CF patients colonized with *C*. *albicans* (Fig 1). As vertical transmission of *C*. *albicans* from mother to child has been previously shown [15], we also isolated 15 maternal strains from the vaginal and/or oropharyngeal areas of 5 mothers (Mothers of patients CF01, CF02, CF07, CF15 and CF20, S1 Table, Fig 1) to determine if *C*. *albicans* strains colonizing the corresponding CF patients could be of maternal origin. Intertestingly, we found that, on many occurrences, individual patients carried strains with identical diploid sequence types (DSTs, see Materials and Methods, Fig 1), suggesting that they were colonized with genetically close strains. Only two patients out of five (CF01 and CF15) carry isolates with DSTs identical to those from their mothers (Fig 1). Notably, ~ half of the patients (8 patients out of 15, CF03, CF05, CF06, CF12, CF15, CF18, CF21 and CF24) carried isolates from clade 4 (Fig 1), including 3 patients (CF03, CF12 and CF15) carrying the same DST (DST95). There is no evidence, so far, pointing to potential strain transmission between these patients; although one could speculate that MoCF15 transmitted the strain to her child (S1 Table).

To better visualize the extent of similarity/divergence between the identified DSTs, we constructed a minimum-spanning tree (S1 Fig). As expected, DSTs belonging to the same clade clustered together, forming in total 3 clonal complexes and 4 singletons representative of six *C*. *albicans* clades (1, 3, 4, 8, 10 and 17) and an unassigned DST (DST228, light blue, S1 Fig). The minimum-spanning tree analysis confirms the prevalence of strains from clade 4 among our set of CF isolates, with a relative predominance of DST95 (S1 Fig). Taken together, our MLST data indicate that each CF patient is colonized with genetically related strains, while a majority of CF patients is colonized with strains from clade 4, a clade enriched with isolates from Middle East Africa [16].

### Serial *C*. *albicans* clinical isolates from patient CF02 display differential ability to filament and form biofilms

Our findings that *C*. *albicans* strains serially recovered from a given CF-patient airway share identifcal DSTs suggest that they are genetically related, which should facilitate comparative analyses between them. Among the 7 CF patients chronically colonized by *C*. *albicans* (CF02, CF03, CF04, CF05, CF06, CF07 and CF12, S1 Table), patient CF02 was the most regularly sampled patient within a 12-month period (*i*.*e*. 8 times every 1.5 month on average, S1 Table and S2 Fig), yielding a set of 18 isolates sharing DST304 (S2 Fig, strain series HBJ1 to HBJ8 isolated from December 2016 to September 2017). Strains were chronologically numbered, according to their sampling date (S2 Fig). Patient CF02 is a pediatric patient with a presumably dynamically-evolving CF airway environment (S2 Table), as he was co-colonized with *C*. *albicans*, *P*. *aeruginosa*, *S*. *aureus* and *Burkholderia cepacia* (S1 and S2 Tables). He displayed pulmonary hyperinflation early after birth, a clinical manifestation in CF closely associated with different types of chronic bronchial infections [17]. We reasoned that a systematic phenotypic analysis of the set of 18 serial isolates from patient CF02 can lead to the identification of phenotypic traits associated with the ability of *C*. *albicans* to thrive more efficiently within the polymicrobial CF host environment.

**Fig 2:**
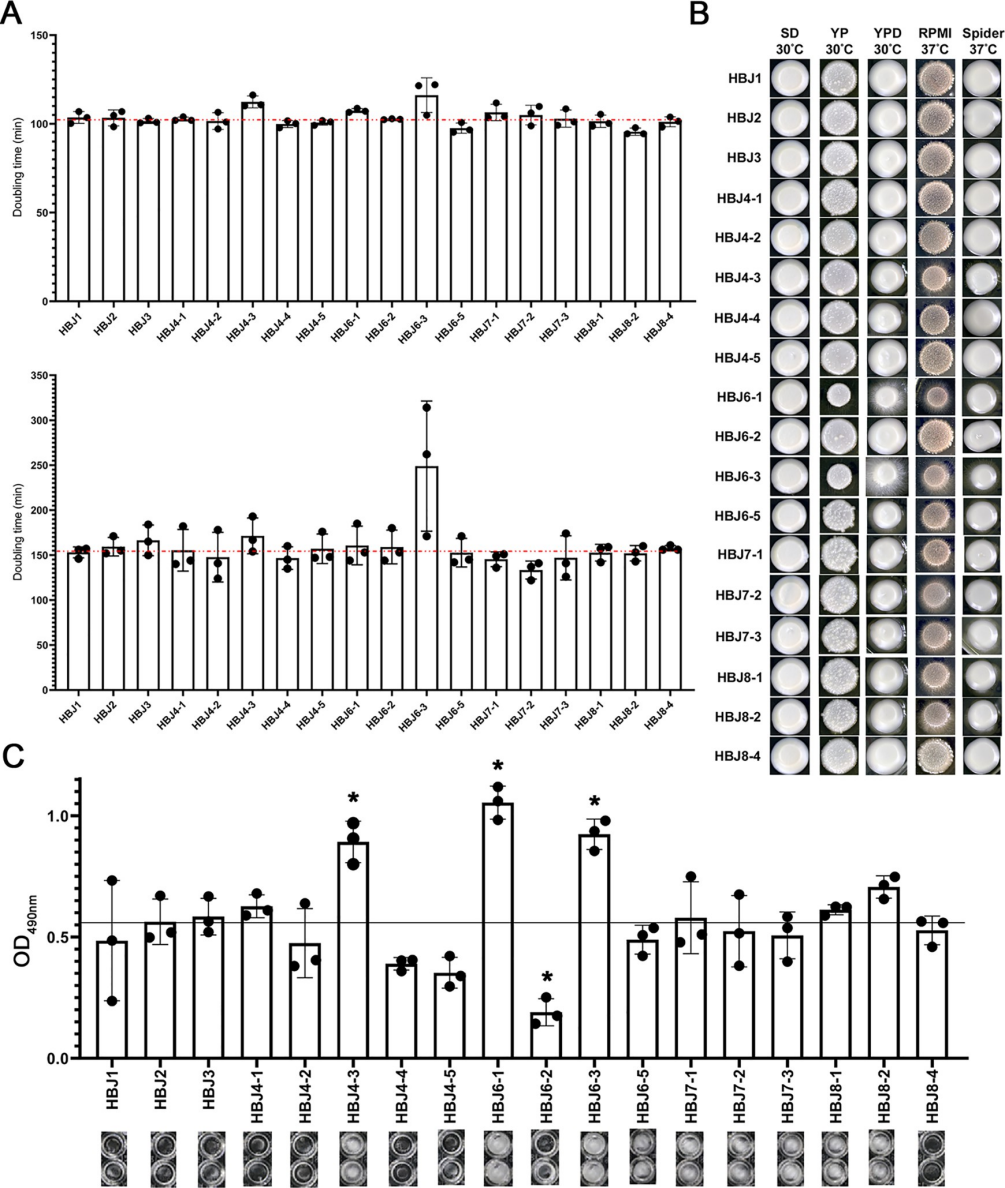
Phenotypic analyses of the complete set of 18 *C*. *albicans* isolates serially recovered from patient CF02. **A.** *C*. *albicans* isolates were individually grown in rich YPD (top panel) and minimal SD (bottom panel) liquid media in 96-well plates at a starting OD_600nm_ of 0.1 in 100 μl of YPD or SD at 30°C. OD_600nm_ was measured every 5 min using a Tecan Infinite 200 reader. Tecan OD_600nm_ readings were converted into “flask OD600nm” readings using the following formula: OD_Flask_ = OD_Tecan_ × 12.2716–1.0543 [95] and doubling times were calculated within the exponential growth interval as previously described [96]. Doubling times (in minutes, average of 3 independent replicates with error bars denoting standard deviations) are indicated on the *y*-axis for each strain. The average growth rate values of strains HBJ6-3 and HBJ4-3 in YPD (top panel) medium differ from the population by at least one standard deviation from the mean. Doubling time of strain HBJ6-1 in YPD medium (top panel) fell short of reaching this threshold. The red horizontal lines mark the median value. **B.** Colony morphology phenotypes of the clinical isolates initially patched (5 μL of a cell dilution at an OD_600nm_ of 0.1) on solid media under filamentation-inducing (RPMI 37°C, Spider 37°C) and -non-inducing (SD at 30°C, YP at 30°C and YPD at 30°C) conditions. **C.** Biofilm formation was measured three times independently by XTT (2,3-Bis-(2-Methoxy-4-Nitro-5-Sulfophenyl)-2H-Tetrazolium-5-Carboxanilide) assay following growth on FBS-precoated polystyrene microtiter plates in rich medium at 37°C as described in Materials and Methods. The average of three independent replicates are shown as OD_490nm_ values on the *y*-axis with error bars denoting standard deviations. Asterisks denote averaged values differing from the population by at least one standard deviation from the mean. The black horizontal line marks the median value. Bottom, photographs of the corresponding two mature biofilms formed independently by the indicated strains on FBS-precoated polystyrene wells, right before quantification by XTT assay.

We first examined strain fitness of the 18 isolates in liquid YPD and synthetic dextrose (SD) media (Fig 2A). In YPD medium, a slight increase in doubling times of strains HBJ4-3, HBJ6-1 and HBJ6-3 was noticed, compared to the remaining isolates (Fig 2A, top panel). In SD medium, the increased doubling time of strain HBJ6-3 was even more pronounced relative to the remaining strains (Fig 2A, bottom panel). The apparent altered fitness defect of strains HBJ4-3, HBJ6-1 and HBJ6-3 could be due to morphological alterations that interfered with OD_600nm_ readings, detectable as an irregular growth curve at stationary phase (S3 Fig, red arrows). We therefore examined colony morphology of the complete set of 18 strains on different solid growth media (Fig 2B). On SD medium at 30°C, all strains displayed smooth, equally-sized, creamy patches (Fig 2B). On YP (deprived of glucose), YPD (both at 30°C), RPMI and Spider (both at 37°C), strains HBJ6-1 and HBJ6-3 displayed a strong hairy/fuzzy morphology (Fig 2B). A strong hairy/fuzzy phenotype was also detected in strain HBJ4-3, on RPMI and Spider media at 37°C (Fig 2B). Because filamentous growth and biofilm formation are interconnected processes in *C*. *albicans*, we tested the ability of the complete set of strains to form biofilms on fetal bovine serum pre-coated polystyrene microtiter plates, at 37°C (Fig 2C, see Materials and Methods). Strikingly, we found that strains HBJ4-3, HBJ6-1 and HBJ6-3 formed biofilms more efficiently than do the remaining isolates (Fig 2C), correlating with their stronger ability to filament/display altered colony morphology on different growth media (Fig 2B). We conclude that a subset of *C*. *albicans* isolates serially recovered from patient CF02 displays enhanced ability to filament and form biofilms.

### Hyperfilamentous strains display enhanced survival in dual-species biofilms formed with *P*. *aeruginosa*

**Fig 3:**
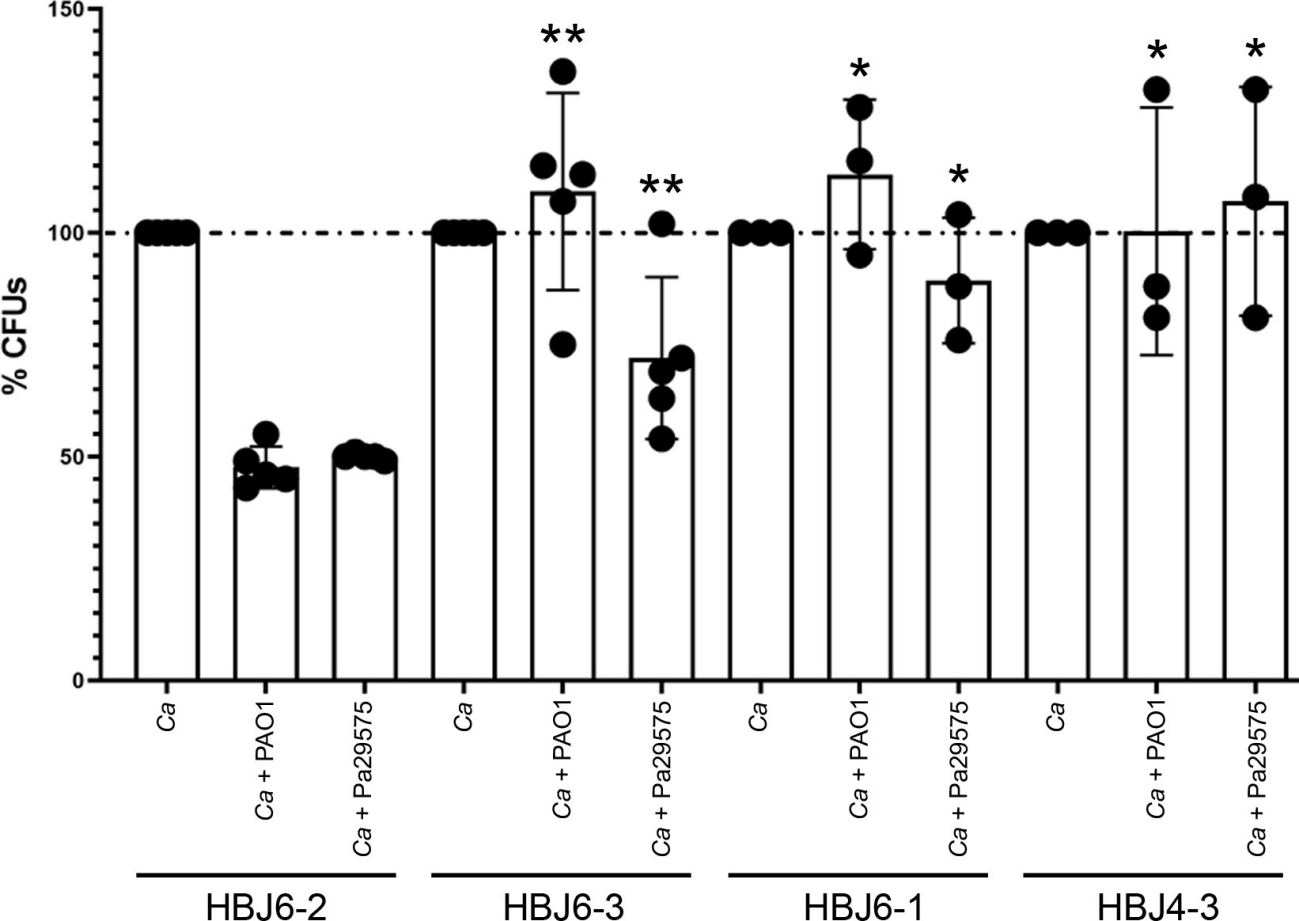
Quantification of the relative survival of *C*. *albicans* CF isolates HBJ6-2, HBJ6-3, HBJ6-1 and HBJ4-3 in dual species biofilms formed with *P*. *aeruginosa*. Preformed 24-h biofilms with either *C*. *albicans* alone (*Ca*, monospecies biofilms made by HBJ6-2, HBJ6-3, HBJ6-1 and HBJ4-3) or both *C*. *albicans* and *P*. *aeruginosa* (dual-species biofilms, *Ca* + PAO1, *Ca* + Pa29575) were treated with DNase I to degrade the extracellular matrix then detached from the surface of wells by scraping (see Materials and Methods). The resulting cell suspensions were diluted and plated onto antibiotic-containing YPD agar to determine the percent CFUs (*y*-axis, %CFUs) of *C*. *albicans* cells co-cultured with *P*. *aeruginosa* PAO1 or Pa29575 relative to biofilm growth of *C*. *albicans* cells alone (*y*-axis, %CFUs set to 100%). The experiments were carried out 3 to 5 times independently and data are plotted as the average of 5 independent biological replicates on the *y*-axis with error bars denoting standard deviations. Statistical analysis was performed using the non-parametric Mann-Whitney test that compares %CFUs for HBJ6-3, HBJ6-1 or HBJ4-3 in each co-incubation condition (*Ca* + PAO1 or *Ca* + Pa29575) with the %CFUs for HBJ6-2 in the corresponding co-incubation condition. *, *P*<0.05; **, *P*<0.01.

Because strains HBJ4-3, HBJ6-1 and HBJ6-3 displayed a stronger ability to filament and form biofilms, we reasoned that, in the context of CF, these strains could more efficiently survive in mixed biofilms formed with *P*. *aeruginosa*. We tested whether the three strains could better compete with *P*. *aeruginosa* in dual-species biofilms, than could the poorly filamentous and weak biofilm-former isolate HBJ6-2 (Fig 3). Strain HBJ6-2 was selected in this assay because it was co-isolated with strains HBJ6-1 and HBJ6-3 from the same sputum sample of patient CF02 (S2 Fig). Strain HBJ6-2 is a weakly filamentous isolate, forming pseudohyphae and/or short hyphae in different filamentation-inducing media (S4 Fig, panel A). We performed a well-established *C*. *albicans*-*P*. *aeruginosa* biofilm assay in microtiter plates at 37°C for 24 h in DMEM medium [14,18,19] (see Materials and Methods). Both HBJ6-2 and the representative hyperfilmentous strain HBJ6-3 could form monospecies biofilms under these conditions (S4 Fig, panel B). We quantified the abundance of HBJ4-3, HBJ6-1, HBJ6-3 and HBJ6-2 cells in dual-species biofilms, relative to their abundance in monospecies biofilms (expressed as % CFUs, Fig 3). To increase clinical relevance, biofilms were grown with *P*. *aeruginosa* clinical isolate Pa29575, a strain co-isolated from patient CF02, in addition to *P*. *aeruginosa* reference strain PAO1 (Fig 3). We strikingly found that the relative abundance of all hyperfilamentous strains, HBJ6-3, HBJ6-1 and HBJ4-3, in dual-species biofilms formed with either *P*. *aerugiona* strains PAO1 or Pa29575 was significantly increased compared to that of poorly filamentous isolate HBJ6-2 (Fig 3), indicating that although the four strains are genetically similar, they differ in their capacity to survive in dual-species biofilms formed with *P*. *aeruginosa*.

### Whole-genome sequencing of serial *C*. *albicans* clinical isolates from patient CF02 confirms their genetic relatedness

**Fig 4:**
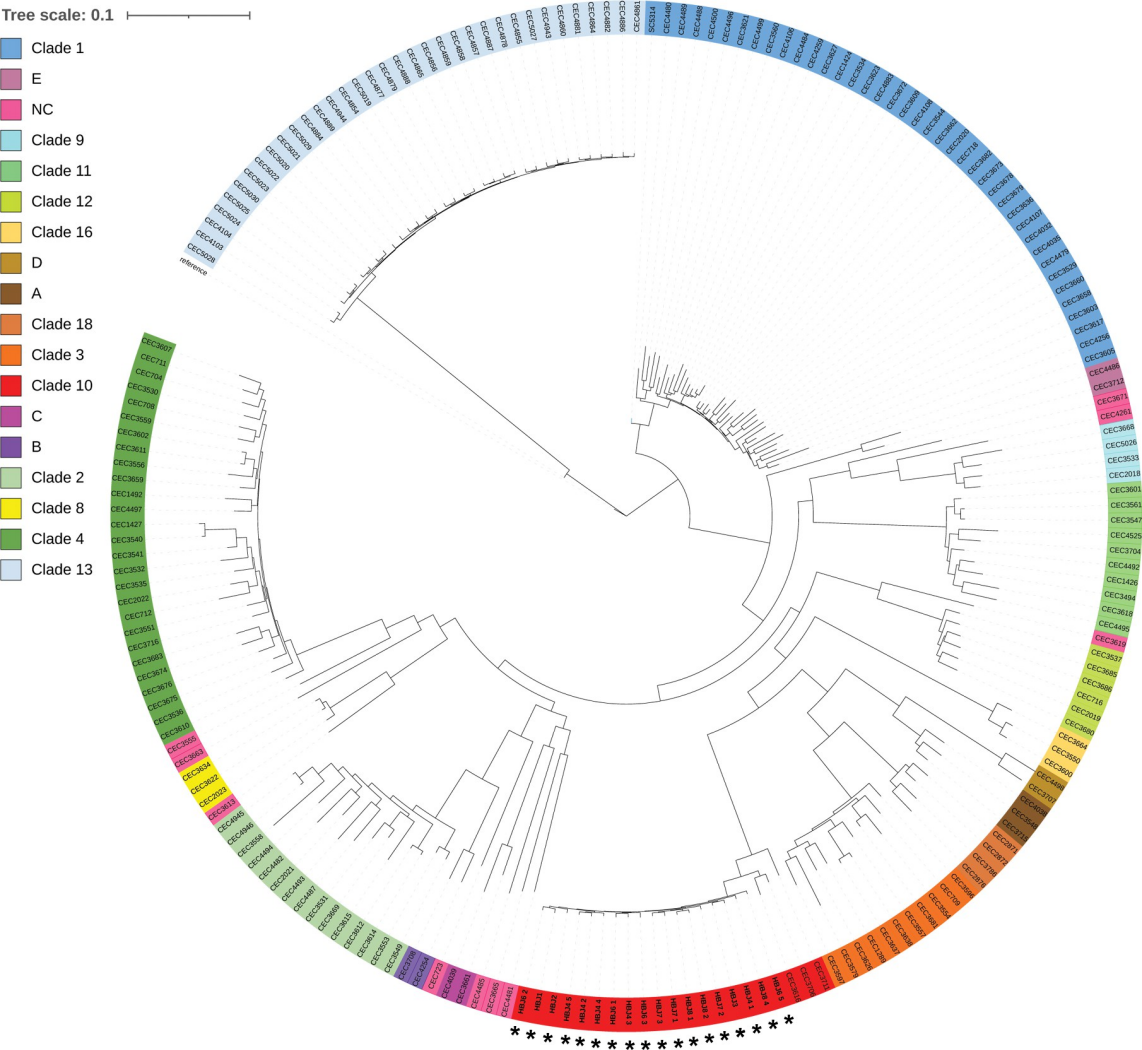
Maximum likelihood phylogenetic tree showing relationships between 200 *C*. *albicans* isolates including those from patient CF02. A total of 200 *C*. *albicans* isolates (182 isolates from Ropars *et al*. together with 18 isolates used in this study, shown in red and indicated by asterisks) were clustered into 17 distinct genetic clades, including 12 clades previously found using multilocus sequencing typing (MLST) and five recent ones (A, B, C, D, and E) described by Ropras *et al*. [20]. The majority of the isolates belonged to clades 1 (n  =  40), 4 (n  =  27), 10 (n = 21), and 13 (n = 35). *C*. *albicans* isolates from patient CF02 (n = 18) were clustered in clade 10 (indicated by asterisks), with a clonal subpopulation structure. Scale bar, 0.1 nucletide substitutions per site on average.

Although MLST is a powerful tool for analyzing the population structure of *C*. *albicans* isolates from multiple host sources and niches of the human body [16] (Fig 1), it does not allow to perform high-resolution and/or in-depth analyses of the genomic alterations that could have occurred within a set of serial isolates from a given patient, which could point to adaptive changes within the host environment. Hence, we subjected the complete set of HBJ isolates from patient CF02 to whole-genome sequencing (WGS) and mapped the resulting reads to the diploid Assembly 22 of the *C*. *albicans* reference genome (strain SC5314, see Materials and Methods). The average sequencing depth was 138×, with a range of 101× to 169×, allowing to robustly determine the extent of genetic variability in our set of isolates. We performed variant calling and filtering analyses, to identify high-confidence single nucleotide polymorphisms (SNPs) and determine the genetic relatedness of the isolates (see Materials and Methods). Compared to the SC5314 reference genome, we found 132,586 highly confident SNPs across the 18 HBJ isolates (S4 Table). Analysis of genetic variation revealed that each of the 18 isolates carried on average 102,148 heterozygous SNPs (versus 65,629 heterozygous SNPs on average in each of 182 clinical isolates collected worldwide [20]), indicating that their genomes were extensively heterozygous (S5 Fig). Mapping of the heterozygous SNPs using a 10-kb sliding window confirmed the genetic relatedness of the 18 serial isolates (S5 Fig), differing from each other by 1,337 SNPs on average (S5 Table). We constructed a phylogenetic tree where they were compared to a set of 182 isolates collected worldwide by Ropars *et al*. [20] (see Materials and Methods, Fig 4). We found that the 18 strains strongly clustered with each other among members of clade 10 (Fig 4, red cluster), displaying a highly clonal subpopulation structure (Fig 4, indicated with asterisks). We verified if copy number variations may have occurred in their genomes, by determining the allele balance at heterozygous sites (ABHet, see Materials and Methods) and plotting ABHet values across the 8 chromosomes (S6 Fig). No copy-number variations were detected in any of the 18 strains (S6 Fig). Taken together, our WGS data confirm the genetic relatedness of the 18 serial isolates recovered from patient CF02 and validate our assumption of their clonal origin.

### A handful number of non-synonymous SNPs differentiate strains HBJ4-3, HBJ6-1 and HBJ6-3 from the remaining CF02 isolates

Although genetically related to each other, the set of 18 serial isolates includes 3 strains (HBJ4-3, HBJ6-1 and HBJ6-3) that phenotypically differ from the remaining isolates. First, the three strains are hyperfilamentous (Fig 2B) and are potent biofilm formers (Fig 2C). Second, they better compete with *P*. *aeruginosa* in dual-species biofilms, compared to the poorly filamentous and weak biofilm-former strain HBJ6-2 (Fig 3). We hypothesize that these strains acquired genetic alterations that led to the emergence of new traits allowing them to better compete with other CF-associated microbes, such as *P*. *aeruginosa*, and better survive in the CF-lung environment. Consequently, we mined the list of 132,586 highly confident SNPs across the 18 HBJ isolates (S4 Table) to identify SNPs that are exclusively shared by the three strong biofilm-former strains HBJ4-3, HBJ6-1 and HBJ6-3 (S6 Table). We found 239 SNPs respecting this criterion, including 154 in protein-coding regions (ORFs and exons), 76 in intergenic regions, 6 within introns and 3 in the major repeat sequence MRS-1 (S6 Table). Among the 154 SNPs in protein-coding sequences, we identified 34 non-synonymous SNPs in 19 ORFs (S7 Table). We reasoned that if non-synonymous SNPs were potentially linked to adaptive growth in the CF lung environment, they should be rarely (if not at all) detected among strains isolated from niches other than the CF airways. We therefore excluded 20 non-synonymous SNPs prevalent among 182 non-CF isolates previously sequenced by Ropars *et al*. [20], narrowing down the list to 14 non-synonymous SNPs in 7 ORFs (Table 1). Interestingly, all 14 non-synonymous SNPs were heterozygous SNPs; half of them were located in orf19.4346, encoding a homolog of *S*. *cerevisiae* Sec16, involved in vesicular trafficking, while two were carried by orf19.427, encoding a homolog of *S*. *cerevisiae* telomeric DNA binding protein Rif1 (Table 1). The remaining 5 non-synonymous SNPs were individually located in 3 uncharacterized ORFs (orf19.415, orf19.2310 and orf19.4406) and 2 ORFs encoding transcription factors of the zinc cluster family, Rob1 and Zfu3 (Table 1). Rob1 is a positive regulator of biofilm formation and filamentous growth in *C*. *albicans* [21–23], whereas Zfu3 is an uncharacterized transcription factor that was previously shown to negatively regulate *C*. *albicans* morphogenesis [24,25]. In conclusion, our analyses identified a handful number of non-synonymous heterozygous SNPs in hyperfilamentous and strong biofilm-former strains HBJ4-3, HBJ6-1 and HBJ6-3 that differentiates them from the remaining CF02 isolates and from 182 non-CF strains collected worldwide. We postulate that a subset of these SNPs could have mediated the emergence of CF-adaptive traits in these isolates.

**Table 1:** List of 14 non-synonymous SNPs found in 7 ORFs that are exclusively shared between hyperfilamentous and strong biofilm former strains HBJ4-3, HBJ6-1 and HBJ6-3, and are not present across 182 non-CF strains previously sequenced by Ropars *et al*. [20].

Position of SNP on chromosome^*a*^	Systematic name^*b*^	orf19#^*c*^	Gene name^*d*^	Description^*e*^	Amino acid change^*f*^
Chr1-1129060	C1_05380C	orf19.427		Ortholog(s) have telomeric DNA binding activity	A1442V
Chr1-1133341					V15A
Chr1-1151886	C1_05490C	orf19.415		Unknown function	A190V
Chr1-2435233	C1_11050W	orf19.2310		Predicted single-stranded nucleic acid binding protein; flow model biofilm induced	I214N
Chr1-2994104	C1_13620W	orf19.4998	*ROB1*	Positive regulator of biofilm formation and filamentous growth	G299E
Chr2-1178684	C2_05770W	orf19.6888	*ZFU3*	Regulator of filamentous growth	A127T
Chr4-1325727	C4_05960W	orf19.4406	*NIF3*	Ortholog of *S*. *cerevisiae* Nif3, rat catheter biofilm repressed	T56A
Chr5-700569	C5_03140C	orf19.4346	*SEC16*	Ortholog(s) have protein-membrane adaptor activity and role in COPII vesicle coating, macroautophagy, protein localization to endoplasmic reticulum exit site	G1768S
Chr5-700620					A1751T
Chr5-700941					I1644V
Chr5-701063					A1603G
Chr5-701069					I1601N
Chr5-701121					P1584A
Chr5-701132					P1580L

^***e***^Position of non-synonymous SNP on the chromosome (Chrx, where x designates chromosome number, followed by position of SNP on the corresponding chromosome) according to the *C*. *albicans* genome Assembly 22, version A22-s07-m01-r57 at the *Candida* Genome Database (CGD)

^***b***^Systematic name of the ORF where the SNP(s) is (are) located, according to Assembly 22 at the CGD.

^***c***^ORF nomenclature according to Assembly 19 at the CGD

^***d***^Gene name according to the CGD.

^***e***^Description or function of the gene according to the CGD or based on published literature.

^***f***^the resulting amino acid (one-letter abbreviation code) change in the deduced protein sequence of the ORF where the SNP(s) is (are) located.

### A single amino-acid change, G299E, in Rob1 is sufficient to drive hyperfilamentation and increased biofilm formation

Our examination of the list of non-synonymous SNPs exclusively shared by the three hyperfilamentous and strong biofilm-former strains HBJ4-3, HBJ6-1 and HBJ6-3 (Table 1) drew our attention to those carried by the transcription factor-encoding genes *ROB1* and *ZFU3* (Table 1). We focused on these two genes for three reasons. First, they encode decision-making proteins (*i*.*e*. transcription factors), that enable an extensive reprogramming of the transcriptome to induce major phenotypic and developmental changes [26]. Second, zinc cluster transcription fators are drivers of evolutionary adaptation in fungi, by acquiring gain-of-function mutations that circumvent environmental pressure [27]. Third, while Rob1 was shown to be part of a tightly-knit transcriptional network that controls biofilm development [22], both Rob1 and Zfu3 were shown to regulate *C*. *albicans* filamentous growth in a haploinsufficient manner [24]. We hypothesized that the two identified SNPs in *ROB1* (G to A substitution at position 962 relative to ATG translation start site of the *ROB1* ORF) and *ZFU3* (G to A substitution at position 489 relative to ATG translation start site of the *ZFU3* ORF), which respectively introduce amino acid changes at positions 299 (Glycine to glutamic acid, G299E) and 127 (Alanine to threonine, A127T) in their deduced protein sequences (Table 1), could be responsible–at least in part–for the acquisition of the hyperfilamentation and strong biofilm formation phenotypes in clinical isolates HBJ4-3, HBJ6-1 and HBJ6-3.

**Fig 5:**
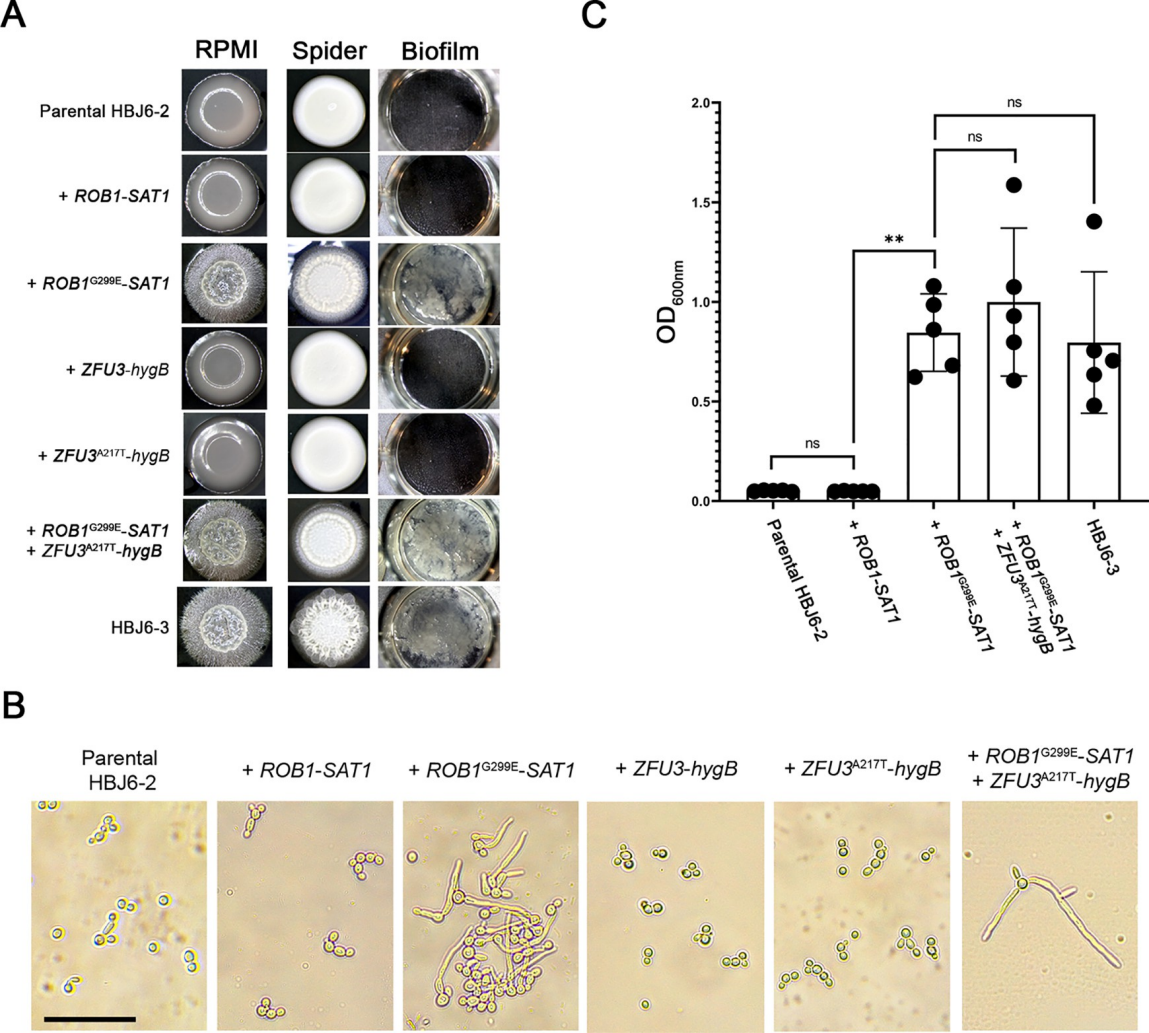
Impact of *ROB1*^G299E^ and *ZFU3*^A217T^ mutations on *C*. *albicans* morphogenesis and biofilm formation. **A.** Representative colony morphology (left and middle columns) and biofilm formation (right column) phenotypes of HBJ6-2 heterozygous mutant derivatives (3 independent clones were generated for each mutant, see S8 Table) carrying the *ROB1*-*SAT1* (+ *ROB1*-*SAT1*, control strain, clone # RCC13, S8 Table), the *ROB1*^G299E^-*SAT1* (+*ROB1*^G299E^-*SAT1*, clone # RC10, S8 Table), the *ZFU3*-*hygB* (+ *ZFU3*-*hygB*, control strain, clone # ZCC5, S8 Table), the *ZFU3*^A217T^-*hygB* (+ *ZFU3*^A217T^-*hygB*, clone # ZC6, S8 Table) and both *ROB1*^G299E^-*SAT1* and *ZFU3*^A217T^-*hygB* (+ *ROB1*^G299E^-*SAT1* + *ZFU3*^A217T^-*hygB*, double mutant, clone # 2MC7, S8 Table) allele replacement cassettes are shown together with those of the parental poorly filamentous HBJ6-2 (Parental HBJ6-2, top row) and the hyperfilamentous HBJ6-3 (bottom row) strains. Strains were patched (5 μl of a cell dilution at an OD_600nm_ of 0.1) on solid media under filamentation-inducing (RPMI, left column; Spider, middle column) conditions and grown at 37°C for 5 days. The indicated strains were also induced to form biofilms for 24 h at 37°C on FBS-precoated polystyrene wells (right columns) as described in panel C. **B.** The strains tested on solid Spider medium in panel A were also tested in liquid Spider medium at 37°C (indicated on top of each image). Overnight, saturated, pre-cultures in YPD medium were diluted to an OD_600nm_ of 0.3 in 2 mL of liquid Spider medium in 12-well polystyrene plates. The diluted cultures were incubated at 37°C under vigorous shaking for 4 hours. The morphology of *C*. *albicans* cells from each culture was examined with a light microscope, at 40× magnification. Scale bar, 100 μm. **C.** Quantitative biofilm formation assay with the parental strain HBJ6-2 (Parental HBJ6-2), the control strain carrying the *ROB1*-*SAT1* allele replacement cassette (+ *ROB1*-*SAT1*), the single *ROB1*^G299E^ mutant (+*ROB1*^G299E^-*SAT1*, clone # RC10, S8 Table), the *ROB1*^G299E^ *ZFU3*^A217T^ double mutant (+ *ROB1*^G299E^-*SAT1* + *ZFU3*^A217T^-*hygB*, clone # 2MC7, S8 Table) and the hyperfilamentous and strong biofilm-former strain HBJ6-3 (HBJ6-3) was performed 5 times independently on FBS-precoated polystyrene microtiter plates in YPD medium at 37°C. An initial incubation for 30 min allowed adherence of cells to the FBS-precoated polystyrene surface, followed by washing and static re-growth in YPD medium at 37°C for 24 h. After a final washing step, biofilm formation was assessed by quantification of biofilm density using spectrophotometry (see Materials and Methods). Averaged OD_600nm_ values from the 5 independent biological replicates are shown on the *y*-axis, with error bars denoting standard deviations. Statistical analysis was performed using the non-parametric Mann-Whitney test. ns, non-significant; **, *P*<0.01.

We used site-directed mutagenesis to introduce the G>A substitution at positions 962 and 489 relative to the ATG translation start site of the *ROB1* and *ZFU3* ORFs, respectively, in the poorly filamentous and weak biofilm-former strain HBJ6-2 (see Materials and Methods). We generated two pairs of allele replacement cassettes, one pair with the dominant selection marker *SAT1* (conferring resistance to nourseothricin) carrying a mutated (*ROB1*^G299E^) or an equivalent wild-type version of *ROB1* (serving as a control); and another pair with the dominant selection marker *hygB* (conferring resistance to hygromycin) harboring a mutated (*ZFU3*^A127T^) or an equivalent wild-type version of *ZFU3* (see Materials and Methods). The four cassettes were used to (co)transform clinical isolate HBJ6-2 and obtain independent single and double heterozygous mutants (together with matching wild-type controls, see Materials and Methods). We tested the ability of the resulting mutants to alter colony morphology, cellular filamentation and biofilm formation (Fig 5). On solid RPMI medium at 37°C, the introduction of the G299E mutation in Rob1 was sufficient to strongly induce a fuzzy phenotype in the resulting heterozygous mutant, phenocopying the hyperfilamentous HBJ6-3 isolate (Fig 5A). Introduction of the A217T mutation in Zfu3, did not affect colony morphology, phenocopying the parental HBJ6-2 strain as well as the control strains carrying wild-type alleles of *ROB1* or *ZFU3* (Fig 5A). We examined the morphology of the heterozygous double mutant and found that it phenocopied both single *ROB1*^G299E^ mutant and strain HBJ6-3 (Fig 5A). Equivalent results were obtained on the filamentation-inducing solid Spider medium at 37°C, albeit with a slightly stronger wrinkling phenotype displayed by strain HBJ6-3 (Fig 5A). We also verified cellular filamentation in liquid Spider medium at 37°C (Fig 5B). While both heterozygous single *ROB1*^G299E^ and double mutants displayed a marked filamentous growth, the *ZFU3*^A217T^ heterozygous mutant failed to do so (Fig 5B), further supporting no role for the *ZFU3*^A217T^ mutation in filamentous growth, at least in a heterozygous context.

Based on the conclusive evidence that the *ROB1*^G299E^ mutation enhances filamentous growth in the poorly filamentous HBJ6-2 strain, we tested its impact on biofilm development (Fig 5, panels A and C). We strikingly found that the heterozygous single *ROB1*^G299E^ mutant developed into a dense biofilm layer on the surface of microtiter-plate wells, phenocopying the strong biofilm-former strain HBJ6-3 (Fig 5A). Again, the heterozygous double mutant was indistinguishable from the single *ROB1*^G299E^ mutant (Fig 5A), reinforcing no contribution of the *ZFU3*^A217T^ heterozygous mutation in biofilm formation. Our results were confirmed with a quantitative biofilm assay, assessing the optical density of biofilm layer at 600 nm (see Materials and Methods, Fig 5C). Both heterozygous single *ROB1*^G299E^ and double mutants strongly formed biofilms at similar levels (Fig 5C). Knowing the function of Rob1 as an activator of filamentous growth and biofilm formation [21–24], we conclude that the G299E substitution in Rob1 acts as a phenotypic gain-of-function mutation, sufficient, in a heterozygous context, to enhance filamentous growth and biofilm development in *C*. *albicans*.

### The G299E mutation in Rob1 induces the expression of Rob1 targets

**Fig 6:**
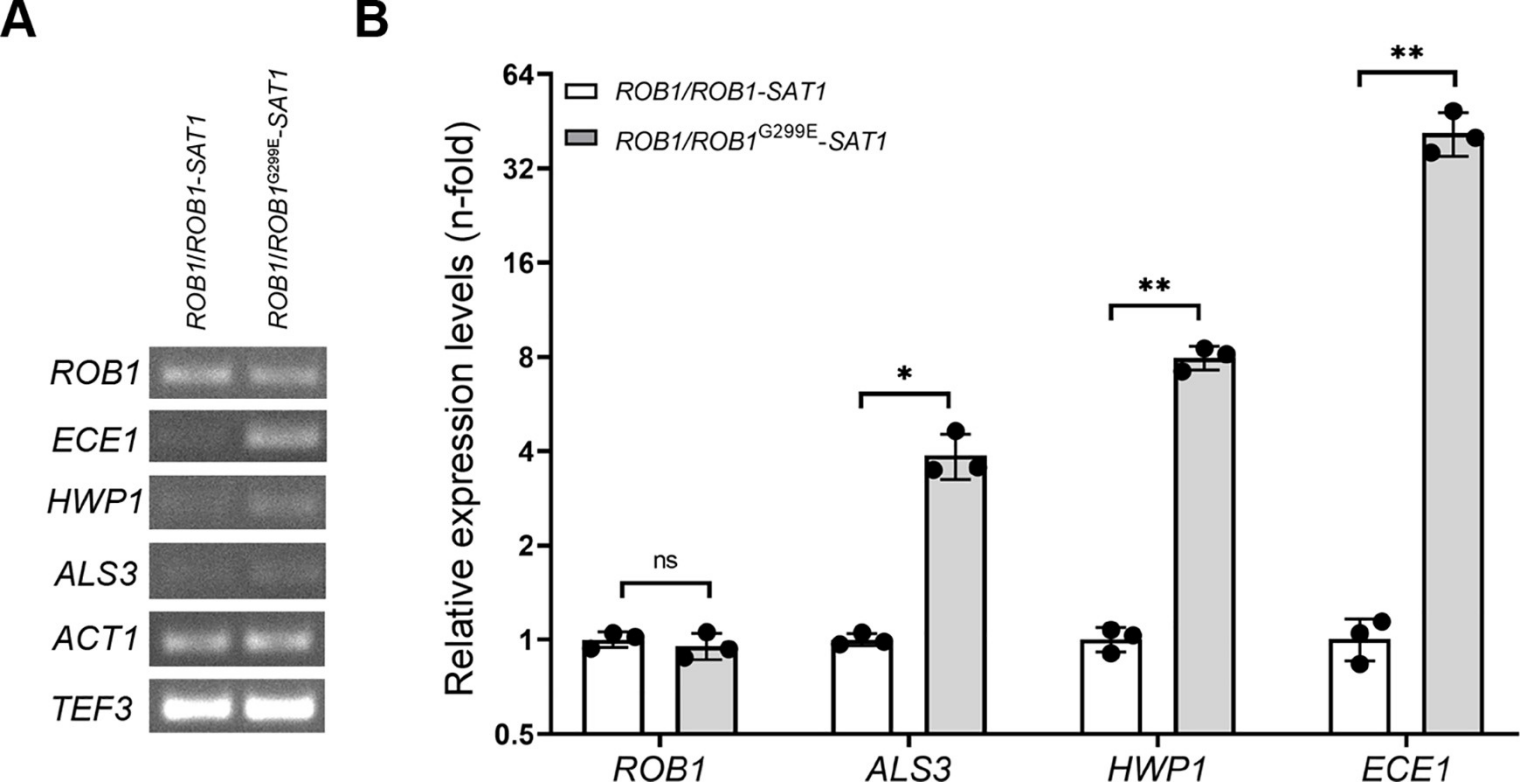
Impact of the *ROB1*^G299E^ mutation on the expression of Rob1 targets. **A.** The expression of *ROB1*, *ECE1*, *HWP1* and *ALS3* in HBJ6-2 strain derivatives *ROB1*/*ROB1*-*SAT1* (left) and *ROB1*/*ROB1*^G299E^-*SAT1* (right) was qualitatively assessed by reverse-transcription polymerase chain reaction (RT-PCR) and agarose gel electrophoresis of the RT-PCR products using specific primers listed in S3 Table. The *ACT1* and *TEF3* loading control amplification signals are shown at the bottom of the panel. **B.** The relative expression of the same genes (*y*-axis, relative expression levels (n-fold)) was also quantitatively analyzed by qRT-PCR. The expression of endogenous gene *ACT1* was used as the normalization standard, and the relative expression of the indicated genes (bottom) in the *ROB1*^G299E^-*SAT1*/*ROB1* mutant (light gray-filled histograms) compared to its expression in the *ROB1*-*SAT1*/*ROB1* (open histograms) was determined using the cycle threshold (ΔΔCt) method using the average ΔCt values of the *ACT1* gene in the *ROB1*-*SAT1*/*ROB1* strain as a calibrator (see Materials and Methods). The assays were carried out three times independently and statistical analysis was performed using a Welch’s *t*-test. ns, not significant; *, *P*<0.05; **, *P*<0.01.

The phenotypic consequences of the G299E substitution in Rob1 suggest that this mutation may convert transcription factor Rob1 into a hyperactive state, hence impacting on transcriptional regulation of Rob1 targets by increasing their expression in the absence of inducing cues, as previously shown with other regulators of this family [26,28]. We tested this hypothesis in HBJ6-2 strain derivatives *ROB1*^G299E^-*SAT1*/*ROB1* and *ROB1*-*SAT1*/*ROB1*—which genetically differ only by the G299E gain-of-function mutation in Rob1 (Fig 6). Both strains were grown in YPD medium at 30°C, then total RNA was extracted and subjected to q/RT-PCR analyses (see Materials and Methods). We qualitatively (Fig 6A) and quantitatively (Fig 6B) assessed the expression levels of Rob1 targets *ALS3*, *HWP1* and *ECE1* [22,29], as well as *ROB1* itself, using specific primers (S3 Table). While *ALS3*, *HWP1* and *ECE1* gene expression levels in the HBJ6-2 derivative *ROB1*-*SAT1*/*ROB1* were undetectable by agarose gel electrophoresis of RT-PCR products, the three genes displayed increased expression levels in the *ROB1*^G299E^-*SAT1*/*ROB1* mutant (Fig 6A). *ROB1* gene expression was detectable at comparable levels in both HBJ6-2 strain derivatives, paralleling with the expression patterns of *ACT1* and *TEF3* loading controls (Fig 6A). Quantitative RT-PCR analyses further confirmed the induction of *ALS3*, *HWP1* and *ECE1* gene-expression in strain *ROB1*^G299E^-*SAT1*/*ROB1* compared to strain *ROB1*-*SAT1*/*ROB1* (Fig 6B), reaching levels as high as 4.7-fold, 8.1-fold and 35.8-fold, respectively (Fig 6B). Taken together, these data demonstrate that the G299E substitution in Rob1 acts as a gain-of-function mutation, leading to strong activation of the expression of Rob1 targets.

### Loss-of-function of *ROB1* abolishes the hyperfilamentation and strong biofilm formation phenotypes in strain HBJ6-3

**Fig 7:**
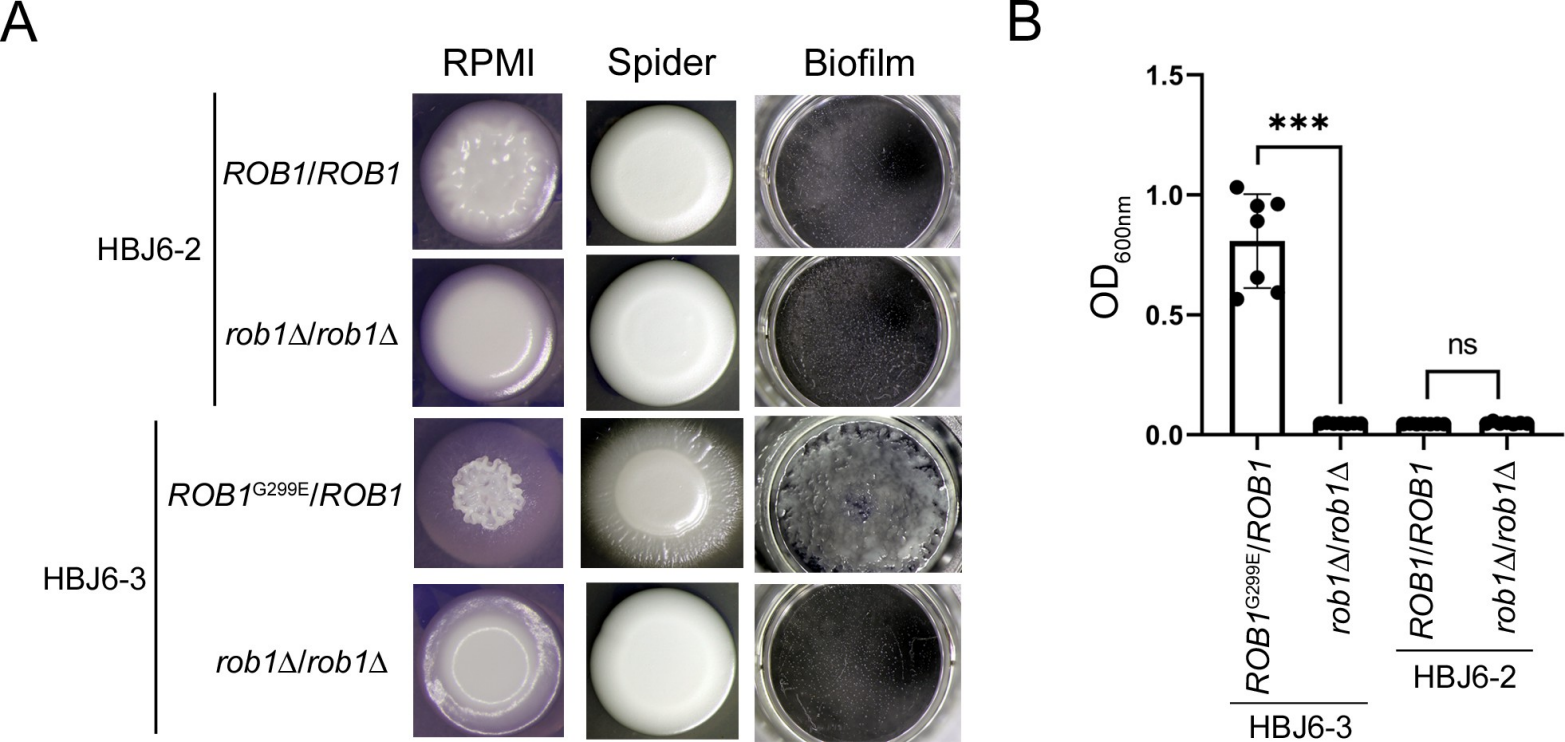
Contribution of *ROB1* to colony morphology and biofilm formation in strain HBJ6-3. **A.** Representative colony morphology (left and middle columns) and biofilm formation (right column) phenotypes of HBJ6-2- and HBJ6-3-derived homozygous *rob1*Δ/*rob1*Δ mutants (5 independent clones were generated from each parental strain, see S8 Table) that were subjected to a CRISPR-Cas9-driven homozygous inactivation of the *ROB1* gene using the dominant selection marker *SAT1* (see Materials and Methods) are shown. The parental poorly filamentous HBJ6-2 strain (*ROB1*/*ROB1*, top row) and the *rob1*Δ/*rob1*Δ mutant derivative (2^nd^ row from top, *rob1*Δ/*rob1*Δ, strain MGY16, clone # KOC28, S8 Table), together with the parental hyperfilamentous strain HBJ6-3 (*ROB1*^G299E^/*ROB1*, third row from top) and the *rob1*Δ/*rob1*Δ mutant derivative (bottom row, *rob1*Δ/*rob1*Δ, strain MGY17, clone # KOC7, S8 Table) were patched (5 μl of a cell dilution at an OD_600nm_ of 0.1) on solid media under filamentation-inducing (RPMI, left column; Spider, middle column) conditions and grown at 37°C for 5 days. The indicated parental strains and their mutant derivatives were also induced to form biofilms for 24 h at 37°C on FBS-precoated polystyrene wells (right columns) as described in panel B. **B.** Quantitative biofilm formation assay was performed 6 times independently on FBS-precoated polystyrene microtiter plates in YPD medium at 37°C with the parental strains and the corresponding *rob1*Δ/*rob1*Δ mutant derivatives described in panel A (shown beneath the *x*-axis). An initial incubation for 30 min allowed adherence of cells to the FBS-precoated polystyrene surface, followed by washing and static re-growth in YPD medium at 37°C for 24 h. After a final washing step, biofilm formation was assessed by quantification of biofilm density using spectrophotometry (see Materials and Methods). Averaged OD_600nm_ values from the 6 independent biological replicates are shown the *y*-axis, with error bars denoting standard deviations. Statistical analysis was performed using the non-parametric Mann-Whitney test. ns, non-significant; ***, *P*<0.001.

Our finding that the gain-of-function mutation G299E in Rob1 converts the poorly filamentous and weak biofilm-former strain HBJ6-2 into a hyperfilamentous and strong biofilm-former mutant fostered us to test whether deletion of *ROB1* in strain HBJ6-3 would lead to the reverse phenotype. It would also assess the contribution of *ROB1* in the ability of HBJ6-3 to hyperfilament and strongly develop into biofilms. We used the transient CRISPR-Cas9-mediated gene deletion strategy [30] to disrupt both copies of *ROB1* in clinical isolates HBJ6-2 and HBJ6-3 (see Materials and Methods, Fig 7). In the poorly filamentous isolate HBJ6-2, disruption of both copies of *ROB1* converted the weak colony wrinkling phenotype observed on RPMI medium, at 37°C, into a smooth colony phenotype in the resulting mutant (Fig 7A, left column). Disruption of *ROB1* in strain HBJ6-3, however, had a dramatic impact on colony morphology; with total loss of the strong colony fuzziness/wrinkling on filamentation-inducing solid media RPMI and Spider, at 37°C (Fig 7A, left and middle columns). The dense biofilm layer formed by strain HBJ6-3 was totally lost in the resulting *rob1*Δ/*rob1*Δ mutant (Fig 7A, right column). A quantitative biofilm assay confirmed our observation (Fig 7B), showing that biofilm density levels of strain HBJ6-3 significantly decreased in the resulting mutant, reaching levels as low as those detectable in both the HBJ6-2 strain and the *rob1*Δ/*rob1*Δ derivative (Fig 7B). Taken together, these data, along with data from Fig 5 indicate that the *ROB1* locus is the genetic driver of the ability of CF02 clinical isolates HBJ4-3, HBJ6-1 and HBJ6-3 to hyperfilament and strongly form biofilms, due to the acquisition of a heterozygous gain-of-function mutation in *ROB1*.

### The *ROB1*^G299E^ mutation is sufficient to confer increased survival of *C*. *albicans* in dual-species biofilms formed with *P*. *aeruginosa*

**Fig 8:**
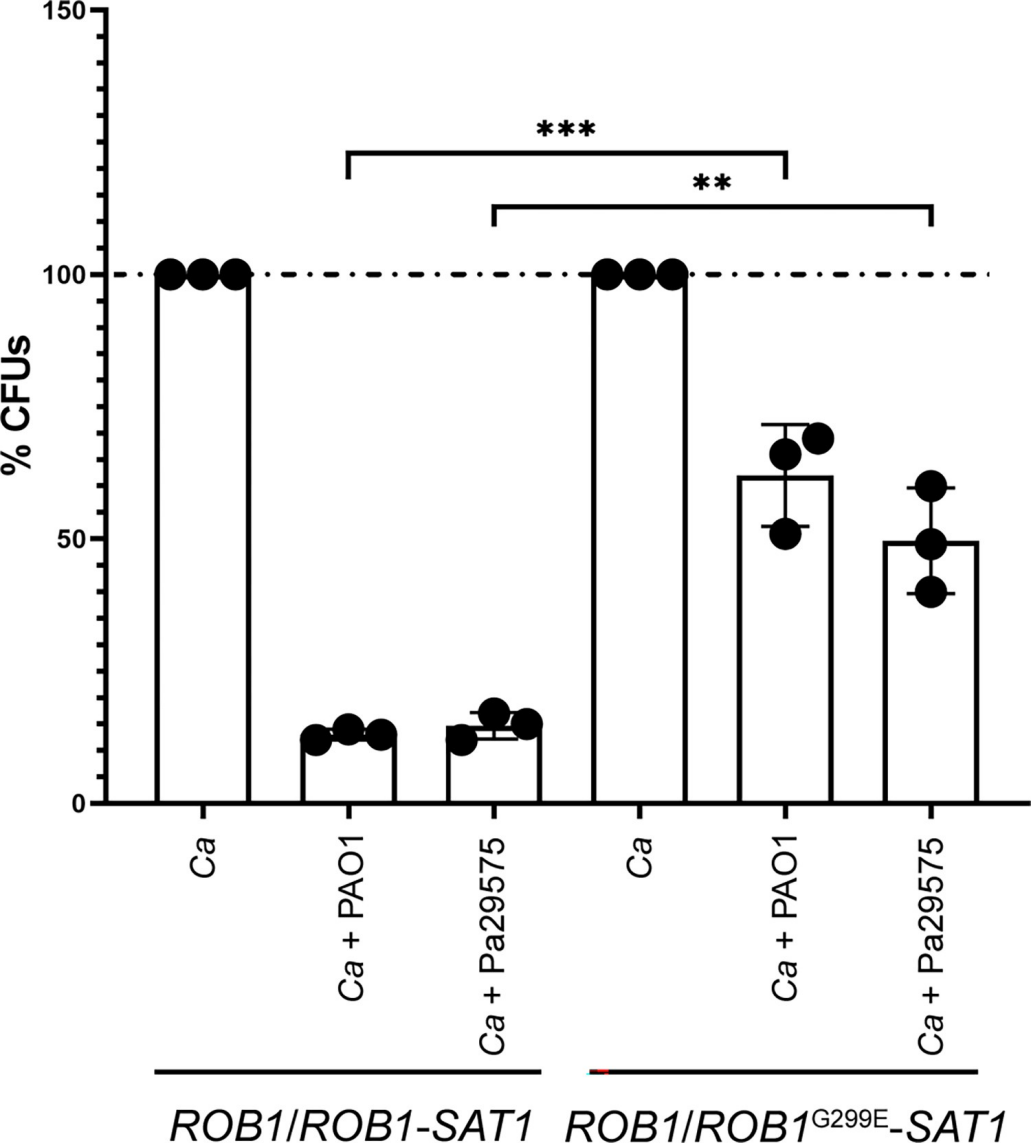
Impact of *ROB1*^G299E^ mutation on survival of *C*. *albicans* in dual-species biofilms formed with *P*. *aeruginosa*. Preformed 24-h biofilms with either *C*. *albicans* alone (monospecies biofilms, *Ca*) or both *C*. *albicans* and *P*. *aeruginosa* (dual-species biofilms, *Ca* + PAO1, *Ca* + Pa29575) were treated with DNase I to degrade the extracellular matrix then detached from the surface of wells by scraping (see Materials and Methods). The resulting cell suspensions were diluted and plated onto antibiotic-containing YPD agar to determine the percent CFUs (*y*-axis, %CFUs) of *C*. *albicans* cells (the HBJ6-2-derived heterozygous control strain *ROB1*/*ROB1*-*SAT1* # RCC13 or the *ROB1*/*ROB1*^G299E^-*SAT1* mutant # RC10, S8 Table) co-cultured with *P*. *aeruginosa* PAO1 or Pa29575 relative to biofilm growth of *C*. *albicans* cells alone (*y*-axis, %CFUs set to 100%). The experiments were carried out three times independently and data are plotted as the average of three independent biological replicates on the *y*-axis with error bars denoting standard deviations. Statistical analysis was performed using a Student’s *t* test. **, *P*<0.01; ***, *P*<0.001.

Because the hyperfilamentous and strong biofilm-former strains HBJ4-3, HBJ6-1 HBJ6-3 were able to better compete with *P*. *aeruginosa* in dual-species biofilms than was the poorly filamentous and weak biofilm-former strain HBJ6-2 (Fig 3), we hypothesized that the *ROB1*^G299E^ mutation conferred such an advantage to these isolates. We therefore challenged both HBJ6-2 strain derivatives *ROB1*^G299E^-*SAT1*/*ROB1* and *ROB1*-*SAT1*/*ROB1*—which genetically differ only by the G299E gain-of-function mutation in Rob1—with *P*. *aeruginosa* strains PAO1 (reference lab strain) and Pa29575 (clinical isolate from patient CF02) in a dual-species biofilm assay (Fig 8). We compared the relative survival of both mutants as described above (Fig 3, see Materials and Methods). We found that the relative abundance of the *ROB1*^G299E^-*SAT1*/*ROB1* mutant in dual-species biofilms formed with either *P*. *aerugiona* strains PAO1 or Pa29575 was significantly increased compared to that of the *ROB1*-*SAT1*/*ROB1* mutant control strain (Fig 8). Our results demonstrate that the *ROB1*^G299E^ mutation is sufficient to confer increased survival of *C*. *albicans* in dual-species biofilms formed with *P*. *aeruginosa*, reflecting its clinical relevance in the context of CF.

## Discussion

In this report, we show that *C*. *albicans* is a chronic colonizer of the lung of patients with CF, an observation found among 7 out of 25 patients (28%) using previously established criteria [6]. Early studies, some of them dating back to the 1970s [31], have shown that *C*. *albicans* can be co-isolated with *P*. *aeruginosa* from the sputum of patients with CF. Consistently, *P*. *aeruginosa* was detected in 5 out of the 7 chronically colonized patients (S1 Table), suggesting that both species potentially engage in polymicrobial interactions within the CF lung environment. Reinforcing this assumption, *P*. *aeruginosa* and *S*. *aureus* were shown to coexist with *Candida* spp. more frequently in sputum samples of CF patients compared with those suffering from other respiratory disorders [4]. We show that most of CF patients individually carry genetically related *C*. *albicans* strains that presumably interact with other classical residents of the CF lung, including *P*. *aeruginosa* (colonizes the airways of patients CF01, CF02, CF03, CF06, CF07, CF11, CF12, CF15 and CF21) and *S*. *aureus* (colonizes the airways of patients CF01, CF02, CF03, CF04, CF05, CF06, CF07, CF11 and CF21) (S1 Table). Our cohort of CF patients is the first to be characterized in terms of the genotypic diversity of *C*. *albicans* strains colonizing their airways. While 8 patients out of 15 carry isolates from clade 4, a clade enriched in strains from the Middle East/Africa [16], three patients (CF03, CF12, CF15) and one mother (MoCF15, mother of patient CF15) were exclusively colonized with strains carrying DST95 (clade 4), the only cases among our cohort of patients sharing the same DST. A previous study performed MLST analyses of 62 *C*. *albicans* strains longitudinally isolated from fecal and tonsillopharyngeal swab samples from 6 pediatric CF patients, 10 children undergoing treatment for cancer and 7 healthy children [32]. Only three of the 23 children were individually colonized with genetically unrelated strains in the longitudinal follow-up [32]. In fact, many reports have documented that the same or closely related strains generally colonize different niches of a given patient over time [33–36]. Strains can evolve through genetic microvariation, documented for multiple *C*. *albicans* isolates from single patients [33,37,38]. However, MLST analyses are poorly resolutive when it comes to detect microevolution events at the whole genome level. Experimental evolution studies, where *C*. *albicans* strains propagated in macrophages [39] and *in vivo* in mouse models of gastrointestinal colonization and systemic infection [40] followed by whole-genome sequencing, identified genome alterations in the evolved strains responsible for phenotypic consequences, including the emergence of new traits [39,40]. Evidence of within-host diversity in *C*. *albicans* has been documented by Sitterlé *et al*., through analysis of variability in multiple clinical isolates taken from the oral cavity of healthy individuals [41]. The study showed that genetically distinct and yet closely related isolates co-exist in a healthy individual [41]. Indeed, 757 to 3,830 SNPs on average were found to differentiate an isolate from another in a given healthy individual [41]. Consistently, we found an average of 1,337 SNPs differentiating each clinical isolate from the other in patient CF02 (S5 Table), yet the set of 18 strains displayed a highly clonal subpopulation structure (Fig 4). We propose that a single strain propagated within the airways of patient CF02, leading to a diversified progeny that accumulated microvariation events over time while colonizing the CF airway environment.

We have shown that a subset of *C*. *albicans* serial isolates from patient CF02 acquired the ability to hyperfilament. The acquisition of this trait in *C*. *albicans* isolates collected from CF patients was previously linked to homozygous loss-of-function mutations in the transcription factor-encoding gene *NRG1*, conferring hyperfilamentation to CF isolates that resist to filamentation-repressing effect of *P*. *aeruginosa* [10]. The *C*. *albicans* hyperfilamentous isolates were rare among the CF isolates collected by Kim *et al*., detected in only 25 out of 1,056 strains isolated from 5 out of 23 patients, and were capable of resisting to the filamentation-repressing activity exerted by the quorum sensing molecule phenazine [10]. In our case, the mechanism underlying the emergence of the hyperfilamentation phenotype was the acquisition of a heterozygous gain-of-function mutation, G299E, in transcription factor Rob1 (Figs 5 and 6). We observed the hyperfilamentous phenotype in series 4 (1 isolate, HBJ4-3, out of 5) and 6 (2 isolates, HBJ6-1 and HBJ6-3, out of 4), but not in later series 7 and 8 (6 isolates in total) that we readily sampled within the three subsequent months (S2 Fig). We speculate that more time is needed for the hyperfilamentous lineage to take over from the parental lineage. Another explanation is that filamentous isolates could engage in mixed biofilms and remain embedded in airway tissues or within the thick CF airway mucus, minimizing frequent recovery from sputum samples. Unfortunately, we were unable to sample patient CF02 for a much longer period of time. Both loss-of-function and gain-of-function mutations in regulators of drug resistance (Mrr1) and iron acquisition (Mrs4) from *Candida lusitaniae* [42,43], as well as activating mutations of the Hog1 pathway in *Aspergillus fumigatus* (Pbs1) [44] were identified in the context of CF, leading to adaptation of these species to microbial and CF host factors. It is striking that the number of *C*. *lusitaniae* genes with non-synonymous SNPs that arose during infection were similar to the number we report here (*i*.*e*. 19 ORFs in both studies) (Table S7 and [43]). This similarity could help identifying conserved pathways that mediate the adaptation of both *Candida* species to the CF airway environment.

Interestingly, another heterozygous gain-of-function mutation in Rob1, L672V, was identified in hyperfilamentous *C*. *albicans* mutants isolated following *in vitro* exposure to *Lactobacillus*-secreted 1-acetyl-β-carboline, a compound preventing *C*. *albicans* from filamenting [45]. We tested whether hyperfilamentation of strain HBJ6-3, attributatble to the *ROB1*^G299E^ mutation, was refractory to *P*. *aeruginosa*-mediated inhibition and found that this was indeed the case (S7 Fig). In the presence of *P*. *aeruginosa* strain PAO1, while pseudohyphal growth of strain HBJ6-2 was inhibited, HBJ6-3 still markedly displayed hyphal form (S7 Fig). This further supports the idea of the *ROB1*^G299E^ mutation impacting on the interplay between *C*. *albicans* and *P*. *aeruginosa*, with functional consequences on how both species interact in the context of CF. Indeed, bacteria and fungi competitively thrive within ecological niches in a variety of ways, communicating through the secretion of small molecules and metabolites, physical interactions and alterations in the composition and function of the environment [46,47]. The interaction between *C*. *albicans* and *P*. *aeruginosa* was extensively investigated by other research groups, and was shown to be quite complex as both synergistic and antagonistic interplays can occur simultaneously [48]. While *C*. *albicans* forms dual species biofilms with *P*. *aeruginosa*, reflecting a stimulatory rather than an inhibitory interaction, *P*. *aeruginosa* kills *C*. *albicans* cells through physical association to *C*. *albicans* hyphae [49] and impairs filamentous growth and biofilm formation through the release of secreted molecules [50]. In the CF lung environment, *C*. *albicans* presumably forms multispecies biofilms facilitated by the presence of hypoxic regions within the static mucus of the CF airways, where the decreased pulmonary function, mucus plugging and oxygen consumption by host neutrophils give rise to regions of anoxia [51–54]. Indeed, the transcriptional response of *C*. *albicans* towards *P*. *aeruginosa* in a dual-species biofilm model was shown to be dominated by a response to hypoxia [55]. Our findings that the Rob1^G299E^ gain-of-function mutation leads to increased survival of *C*. *albicans* cells grown in dual-species biofilms with *P*. *aeruginosa* (Fig 8) and confers resistance to filamentation-repressing activity exerted by *P*. *aeruginosa* (S7 Fig) provide evidence in favor of a selective advantage conferred by the G299E mutation in modulating an important *C*. *albicans* fitness attribute in the polymicrobial context of CF. Nevertheless, although the use of a static model of *C*. *albicans*-*P*. *aeruginosa* biofilm formation where both species are statically co-incubated in growth-optimized medium in 96-well polystyrene plates (S4 Fig, panel B) is quite popular [56], it does not take into account the complexity of the components present in the CF sputum/mucus and the shear flow conditions of specific niches of the lung [57]. However, it may lead to rapid depletion of oxygen and formation of microaerophilic to hypoxic biofilms, similar to the biofilms formed in the lungs of CF patients [58]. O’Brien *et al* used a relevant continuous-flow *in vitro* culture model, which enables long-term co-culture of *C*. *albicans* and *P*. *aeruginosa* [59]. They used artificial sputum as a growth medium, which mimics growth of microbes in the CF lung habitat and is known to physiologically recapitulate the nutritional composition of the CF airway secretions [60]. Using an optically clear derivative of artificial sputum medium that allows to microscopically examine differentially fluorescently-labeled *C*. *albicans* and *P*. *aeruginosa* cells in dual species bioflims, Kasetty *et al*. showed that both species accumulate greater biomass under flow [61], in line with previous findings showing that biofilms formed under shear are more compacted and physically robust relative to those grown in static conditions [62]. Such co-culture models could provide the basis for future investigations allowing to test, in different conditions, the contribution of Rob1^G299E^ in *C*. *albicans*’ ability to more efficiently interact with *P*. *aeruginosa* as well as with other CF-associated pathogens, such as *S*. *aureus*.

Rob1 appears to be restricted to species closely related to *C*. *albicans*, namely *C*. *dubliniensis* and *C*. *tropicalis*, and is apparently absent from *C*. *parapsilosis* [63]. While Rob1 does regulate biofilm formation in *C*. *albicans* and *C*. *dubliniensis*, it appears to be dispensable for this process in *C*. *tropicalis* [64,65] and its function may have been rewired in this species. Interestingly, the *Candida* Gene Order Browser (http://cgob.ucd.ie/) [66,67] indicates the presence of syntenic orthologs in the xylose-fermenting CUG-clade species *Scheffersomyces stipitis* and *Spathaspora passalidarum*, which are also known to colonize the gut of woodboring beetles [68]. It is tempting to speculate that Rob1 may have evolved in a subset of CUG-clade species to regulate processes pertaining to microbial interaction/competition in different host niches. Rob1 is a member of the zinc cluster family of transcriptional regulators (ZCFs) that are considered as drivers of evolutionary adaptation in fungi, by acquiring gain-of-function mutations circumventing environmental pressure [27]. As a subfamily of zinc finger proteins [69], ZCFs form one of the largest classes of fungal transcriptional regulators and are exclusively found in fungi and amoeba [28]. Rob1 (991 aminoacids) is predicted to carry a typical N-terminal zinc binuclear cluster DNA binding domain, located between aminoacids 11–54, a middle homology region (MHR, residues 492–561), thought to play a regulatory role in a subset of ZCFs [70], and two predicted C-terminal coiled coil domains (residues 758–838 and 894–939) shown to mediate homo- or heterodimerization in many ZCFs [71–73]. The G299E mutation identified in our study is located between the predicted DNA-binding domain and the MHR of Rob1, whereas the L672V mutation selected during *in vitro* exposure of *C*. *albicans* to the *Lactobacillus*-secreted filamentation-repressing molecule 1-acetyl-β-carboline [45] is located between the predicted coiled coil domains and the MHR. Interestingly, the experimental evolution study performed by MacAlpine *et al* was carried out in the SN95 strain background [45], a derivative of reference strain SC5314 [74] which has been recently shown to naturally carry another heterozygous gain-of-function mutation in Rob1, P946S, explaining why SC5314 is among the most highly filamentous and invasive strains characterized to date [29]. The P946S mutation is located downstream of the predicted coiled coil domain of Rob1 and was also shown to activate the expression of Rob1 target genes *ALS3*, *ECE1* and *HWP1* [29]. As previously described for ZCFs that regulate multidrug resistance in *C*. *albicans* (Tac1, Mrr1, Upc2) [75–77], *C*. *glabrata* (Pdr1) and *S*. *cerevisiae* (Pdr1 and Pdr3) [78,79], the different locations of the three gain-of-function mutations in Rob1 are in line with the notion that ZCF activating mutations are not necessarily confined within key regulatory regions, such as the transactivation domain or the MHR. In fact, the physiological mechanisms of transcriptional activation by ZCFs usually include intramolecular conformational changes occurring upon direct binding of ligand molecules [80–83]. Many ZCFs act as direct sensors of small signaling molecules that include nutrients, metabolic intermediates, antifungal compounds, chemicals and other molecules such as heme [84–88]. The mechanisms whereby mutations in ZCFs convert these proteins into a hyperactive state are not fully understood, but they may lead to induction of conformational changes that lock the transcription factor into a constitutively active status, reminiscent of continuous activation by ligands. Indeed, it was shown that an *S*. *cerevisiae* strain carrying an activating mutation in Pdr1 (F815S) exhibits enhanced occupancy of coactivator complexes at the *PDR5* promoter (one of Pdr1 targets), accompanied by loss of contact between histones and DNA, as well as altered chromatin structure at both promoter and coding sequences of *PDR5* [89]. The nature of the G299E mutation in Rob1 (gain of a negative charge) is reminiscent of mutations that increase the acidity of the trans-activating domain of *S*. *cerevisiae* Gal4, thereby promoting enhanced recruitment of the transcriptional machinery to the target promoters [90]. Alternatively, they could disrupt the interaction of Rob1 with a repressor as previously described for *S*. *cerevisiae* Upc2 involved in sterol homeostasis, where any amino-acid substitution larger than alanine at position G888 converts the protein into a hyperactive state [91]. The frequent occurrence of gain-of-function mutations in ZCFs suggests that a generic mechanism of transcriptional activation exists among the members of this family that remains to be elucidated.

## Materials and methods

### Ethics statement

All procedures complied with ethical standards for human investigations and the principles of the Declaration of Helsinki. Written informed consent for minors was obtained from the patient parent or legal guardian. In case of sampling of family relatives or CF patients aged ≥18 years, an informed consent was signed. The present study protocol was reviewed and approved by the Ethics Committee at Institut Pasteur de Tunis (Application file # 2019/22/I/ LR16IPT/V3).

### CF-patient clinical data and sputum sample collection

Twenty-five antifungal-naive CF patients were recruited in this study (S1 Table). Confirmed CF diagnosis was established based on clinical symptoms with two positive sweat chloride tests, molecular analysis of the CFTR gene and identification of the CF-causing mutation (S2 Table). Data collected includes patient age, gender, CFTR mutation, body mass index, chronic lung colonization with *P*. *aeruginosa*, mucoid phenotype of *P*. *aeruginosa*, allergic bronchopulmonary aspergillosis, bronchiectasis, the presence of *B*. *cepacia*, exacerbation frequency and hepatic cirrhosis (S1 and S2 Tables). Sputum samples were collected as part of routine clinical care from all CF patients, with the assistance of a medical practitioner. In total, 119 sputum samples and one tongue swab sample were obtained over a period of ~2.5 years (December 2016 to April 2019, S1 Table). The studied population was comprised of 23 pediatric and 2 adult patients, with an age range encompassing 2–22 years, the median age being 5 years (S1 Table). The number of samples per patient depended on the frequency of the visits to the BHCHT, Tunisia (S1 Table). We also collected samples from 19 mothers of CF patients, including 19 oropharyngeal swab samples and 3 sputum samples to test for the presence of *C*. *albicans* and assess potential mother-to-child transmission (S1 Table).

### Microbiological method for the isolation and identification of *Candida* spp

The collection of sputum specimens was performed at Children’s Hospital in Tunis. To prevent microbiological contamination of the expectorated sputa, all patients were asked to rinse their mouth twice with water then cough in order to expectorate sputum into a sterile container. Collected specimens from subjects were solubilized by dilution into normal saline (0.9% NaCl), then plated onto sabouraud dextrose agar, containing chloramphenicol (100 μg/ml) to inhibit the growth of bacteria and cycloheximide (50 μg/ml) to inhibit the growth of saprophytic and growth medium-contaminating fungi, then incubated at 30°C for one week. The isolated yeast species were tested for their ability to form germ tubes and chlamydospores. Only isolates capable of chlamydospore formation were streaked onto chromagar (chromID® *Candida* biomérieux, France) and incubated at 37°C for 5 days. Single blueish colonies (*C*. *albicans* or *C*. *dublinensis*) were restreaked on YPD agar medium then subjected to species identification using two independent approaches: i) Internal transcribed spacer 2 (ITS2) amplification using primer pair 3271-ITS2F and 3271-ITS2R [92] (S3 Table) followed by sequencing with an ABI prism 3500 genetic analyzer (Applied Biosystems) and/or 2) Matrix-Assisted Laser Desorption/Ionization-Time of Flight (MALDI-ToF) biotyper at hôpital Necker-Enfants malades in Paris, France (S1 Table). In total, 146 *C*. *albicans* strains were isolated from 15 patients (S1 Table). Patients with three or more consecutive growth of *C*. *albicans* in sputum cultures or in ≥ 50% of cultures obtained within a 12-month period were considered to have chronic *C*. *albicans* colonization, those with positive cultures in 25–49% of sputum samples obtained within 12 months were classified as having intermittent colonization, and those with no positive cultures or positive findings in < 25% of cultures obtained over 12 months were defined as not colonized [6]. Glycerol stocks were prepared for each identified isolate and stored at -80 C.

### Genotyping of *C*. *albicans* clinical isolates by Multilocus Sequence Typing

MLST of *C*. *albicans* isolates involves the PCR amplification and DNA sequence analysis of 300–400 bp regions from seven housekeeping genes (*AAT1a*, *ACC1*, *ADP1*, *PMI1b*, *SYA1*, *VPS13*, and *ZWF1b*) that are under stabilising selection pressure [93]. MLST data are represented by genotype numbers, which define unique sequences for pairs of alleles, and diploid sequence types (DSTs), which define unique combinations of genotypes. Total genomic DNA of *C*. *albicans* clinical isolates was extracted using a rapid miniprep protocol described previously by Liu *et al*. [94]. The extracted DNA was subjected to PCR amplification of the seven housekeeping genes with primers listed in S3 Table. The nucleotide polymorphisms of the resulting sequences were used for MLST analysis as described by Bougnoux *et al*. [93]. Shortly, PCR was carried out in 50-μl reaction volume containing 2 μl of genomic DNA (10 ng/μl), 1 μl of each primer (0.2 μM), 1 unit of Taq DNA polymerase (Thermo Fisher Scientific), 5 μl of 10X reaction buffer, 5 μl MgCl_2_ (2.5mM) and 4 μL of deoxynucleoside triphosphate (2mM). The PCR products of the seven housekeeping genes were sequenced using the ABI PRISM BigDye Terminator v3.1 sequencing kit according to the manufacturer’s instructions, then runned on an ABI prism 3500 genetic analyzer sequencer (Applied Biosystems). The resulting FASTA files were uploaded to the *C*. *albicans* MLST database (https://pubmlst.org/organisms/candida-albicans) for DST identification.

### Phylogenetic analysis of MLST data and generation of minimum spanning tree

The resulting set of FASTA sequences were concatenated and a phylogenetic tree was constructed by the unweighted-pair group method using average linkages (UPGMA) with 1,000 replicates for bootstrap testing (MEGA X and/or BioNumerics version 8.1.1). Each clade was identified by using the goeBURST version 1.2.1. Briefly, 4.175 DSTs were downloaded from the MLST database as of July 2022, then compared to those of CF isolates by using eBURST algorithm. The DSTs were divided according to an optimal solution for the placement of links between the different DSTs and the organization of strains into clonal clusters. Clades were assigned according to the cluster distribution and the placement of each founder strain of the different clonal complexes. To elaborate the population structure and link together the more closely related isolates originating from different CF patients, a Minimum spanning tree was generated based on the MLST data and the goeBurst cluster output. The analysis was performed using BioNumerics version 8.1.1 (Applied Maths).

### Media and growth conditions

*C*. *albicans* isolates were routinely grown in YPD medium (1% yeast extract, 2% peptone, 2% dextrose) at 30°C under shaking, unless stated otherwise. Agar in a final concentration of 2% was supplemented for medium solidification. To test the ability of *C*. *albicans* clinical isolates to filament, colony morphologies were examined on YPD, YP (without dextrose), SD (6.7 g/L Yeast Nitrogen Base without amino acids and ammonium sulfate, 2% dextrose), Spider (1% peptone, 1% yeast extract, 1% manitol, 0.5% NaCl, and 0.2% K_2_HPO_4_) and RPMI 1640 + GlutaMAX (gibco) supplemented with 50 mM HEPES buffer (Sigma) agar media. Each clinical isolate was diluted to an optical density at 600 nm (OD_600nm_) of 0.1, spotted onto the agar plate then incubated at 37°C for 3 to 5 days to assess colony morphology. Colonies were photographed with a 10× magnification using an EOS 550D camera (Canon, Tokyo, Japan) connected to a Leica M80 stereomicroscope (Leica Microsystems, Rueil-Malmaison, France).

### Strain fitness assay and quantification of doubling times

To monitor for growth defects among CF isolates, growth curves were generated using a TECAN Sunrise device by measuring the OD_600nm_ every 10 min for 42 h (S3 Fig). Strains were individually grown three-times independently in 96-well plates at a starting OD_600nm_ of 0.1 in 100 μl of YPD or SD. The temperature was set at 30°C. TECAN OD_600nm_ readings were converted into “flask OD_600nm_” reading using the following formula: OD_Flask_ = OD_Tecan_ × 12.2716–1.0543 [95] and doubling times were calculated within the exponential growth interval as previously described [96]. The resulting data were analyzed using GraphPad Prism 8.0.2.

### *C*. *albicans* biofilm growth assays

Biofilm growth assays were performed using two methods, the standard optical density assay method [21] and the XTT assay method [97]. The standard optical density assay was performed in 24-well polystyrene plates. Plates were pretreated overnight with fetal bovine serum at 37°C (FBS, gibco, catalog # 10270–106). The following day, each well was inoculated with 2 ml of cells at an OD_600nm_ of 0.3 and incubated at 37°C for 90 min at 110 rpm to allow cell adherence. After 90 min, the plates were washed with 2 ml of 1X phosphate-buffered saline (PBS) to eliminate non-adhered cells and replaced with 2 ml of fresh YPD. Plates were sealed with breathseal sealing membranes (Greiner bio-one) and incubated at 37°C for 24 h with shaking at 110 rpm. The medium was aspirated carefully, and each well was washed twice with 1 ml of 1X PBS. The OD_600nm_ was read on a Tecan Spark multimode reader (Tecan Systems). Sixty-one measurement points at independent locations in each well of the 24-well plate were performed and a heatmap was generated for each well. The average density for each strain was calculated from three independent experiments after subtracting the background value of the control well with YPD only. Biofilm layer was also photographed using an EOS 550D camera (Canon, Tokyo, Japan).

The XTT assay method [97] was performed in FBS-precoated polystyrene 96-well microtiter plates (TPP 96-Well, Flat-Bottom Microplate) in YPD medium at 37°C then cell viability was assessed using the tetrazolium salt (2,3-bis(2-methoxy-4-nitro-5-sulfophenyl)-5-[(phenylamino)carbonyl]-2H-tetrazolium hydroxide (XTT) (Sigma) colorimetric method to detect cellular metabolic activities within *C*. *albicans* biofilms. Strains were grown overnight in YPD at 30°C under shaking. The following day, 100 μl of cell suspension containing 10^6^ cells/ml in YPD medium were pipetted into wells of the 96-well flat-bottom microplate plates and incubated at 37°C for 90 min to allow cell adhesion on the surface of wells. After the incubation phase, YPD medium was aspirated off and wells were washed twice with PBS to eliminate non-adherent cells, then fresh YPD was added. The plates were covered with BREATHseal sealing membranes (Greiner bio-one) and incubated for 24 h. XTT solution was prepared at 0.5 g/L in PBS and filter sterilized with a 0.22 μm filter, aliquoted, and conserved at –70°C for later use. Prior to using, XTT aliquot was thawed out and a stock solution of 10 mM menadione (ChemCruz) was dispensed to a final concentration of 1 μM, then a volume of 100 μl aliquot of XTT/menadione was added to each pre-washed well. The plates were wrapped with aluminum foil as XTT is sensitive to light exposure and incubated for 1 h at 37°C. The colorimetric shift was read at 490 nm on a Tecan Spark multimode reader (Tecan Systems).

### Bacterial strains used in this study

*P*. *aeruginosa* reference strain PAO1 (a gift from Laurent Debarbieux, Institut Pasteur, Paris, France) and clinical strain Pa29575 isolated from patient CF02 were used for dual-species biofilm assay. Prior to each experiment, the bacterial strains were revived from frozen stocks at -80°C by streaking on Luria Bertani (LB) plates and left for overnight incubation at 37°C. Bacterial colonies were then inoculated at 37°C and incubated on an orbital shaker at 200 rpm overnight with aeration prior to co-culture assays with *C*. *albicans*.

### Dual-species biofilms of *C*. *albicans* and *P*. *aeruginosa*

The protocol for *C*. *albicans*-*P*. *aeruginosa* biofilm assay was kindly provided by Dr. Rebecca Hall. Assays were performed as described by Alam *et al*. [14,18,19] with slight modifications. Briefly, *C*. *albicans* was grown overnight in YPD and *P*. *aeruginosa* was grown overnight in LB, and cultures were washed in PBS. *C*. *albicans* strains were resuspended at 1 × 10^6^ cells/mL and *P*. *aeruginosa* strains were resuspended to an OD_600nm_ of 0.2 in Dulbecco Modified Eagle Medium (DMEM, Sigma) supplemented with 1% L-glutamine. Each well of 96-well plates contained 100 μL (1×10^5^ cells) *C*. *albicans* and 10 μL (2.5×10^6^ cells) of *P*. *aeruginosa* (final ratio fungus:bacterium = 1:25). Plates were incubated at 37°C for 2 h to allow cells to adhere, at which point the medium was replaced with fresh sterile medium, and plates incubated statically at 37°C for 24 h. Medium was replaced with 100 μL PBS containing 50 μg/mL DNase I and plates were incubated at 37°C for 1 h to degrade the extracellular matrix. Biofilms were detached from the plates by scraping, serially diluted, and plated onto YPD agar supplemented with 100 μg/mL tetracycline to determine viable cells of *C*. *albicans* (colony forming units, CFUs). Percent CFUs of *C*. *albicans* cells co-cultured with *P*. *aeruginosa* PAO1 or Pa29575 was calculated relative to biofilm growth of *C*. *albicans* cells alone. The experiments were carried out 5 times independently. Statistical analysis was performed using the Mann-Whitney test.

### Whole genome sequencing, variant-calling and phylogenetic analyses

Genomic DNA was prepared according to the method by Amberg *et al*. [98] from a set of 18 *C*. *albicans* clinical isolates (HBJ series, patient CF02, S2 Fig). Whole genome sequencing libraries were prepared at the Biomics core facility [99] at Institut Pasteur, Paris, France, using the Illumina TruSeq™ DNA PCR-Free Low Throughput Library Prep Kit (FC-121-3001) following the manufacturer’s instructions. Libraries were sequenced on an Illumina NovaSeq 6000 device, generating 7 to 12 million 150-bp paired-end reads. Reads have been deposited at the NCBI Sequence Read Archive under BioProject ID PRJNA1008086.

Sequences and genomic variations were analysed essentially as described by Ropars *et al*. [20] and Marton *et al*. [100]. Briefly, each set of paired-end reads was mapped against the *C*. *albicans* reference genome SC5314 haplotype A or haplotype B [101] downloaded from the *Candida* Genome Database (version A22-s07-m01-r57) [102] using Minimap2 version 2.9 [103]. SAMtools, version 1.9, and Picard tools, version 2.8.1 (http://broadinstitute.github.io/picard), were then used to filter, sort, and convert SAM files. SNPs were called using the Genome Analysis Toolkit (GATK) version 3.6, according to GATK best practices [104]. SNPs were filtered using the following parameters: VariantFiltration, QD < 2.0, LowQD, ReadPosRankSum < −8.0, LowRankSum, FS > 60.0, HightFS, MQRankSum < −12.5, MQRankSum, MQ < 40.0, LowMQ, HaplotypeScore > 13.0, HaploScore. Coverages were also calculated using GATK. Besides passing GATK’s filters, we also checked for read depth (it had to be between 0.5 and 1.5 of the mean genome coverage). Highly confident SNPs were displayed on Integrative Genomics Viewer version 2.9.4. [105] by creating a bed file with color-coding of SNPs, enabling navigation on the *C*. *albicans* genome and examination of SNPs in candidate genes. The GATK variant filtration walker (VariantAnnotator) was used to add allele balance information to.vcf files. The value of allele balance at heterozygous sites (ABHet) is a number that varies between 0 and 1. ABHet is calculated as the number of reference reads from individuals with heterozygous genotypes divided by the total number of reads from such individuals. A diploid genome will be defined by an ABHet value of 0.5. A triploid strain may contain either three identical alleles (an allelic frequency of 1) or two identical alleles and one different allele (frequencies of 0.66 and 0.33). A tetraploid strain may have allelic frequencies of either 0.5 (2×2 identical alleles), 1 (4 identical alleles), or 0.25 and 0.75 (3 identical alleles and 1 different allele). Heterozygous SNP density maps were constructed as described by Loll-Krippleber *et al*. [106], by determining the number of heterozygous positions per 10-kb region and plotting each value using Python.

We used RAxML (Randomized Axelerated Maximum Likelihood), a tool for phylogenetic analysis and post-analysis of large phylogenies, to construct phylogenetic relationships between 200 *C*. *albicans* isolates based on highly confident SNPs identified in each of 182 strains from Ropars *et al*. [20] and the 18 isolates from patient CF02, with 1,000 bootstrap replicates. The final circular maximum likelihood phylogenetic tree output was generated using iTOL for the annotation and display of isolates that were color-coded by clade.

### *C*. *albicans* transformation

*C*. *albicans* cells were transformed using the standard lithium acetate procedure, as described previously [107], with minor modifications. Briefly, an overnight culture of *C*. *albicans* was diluted to an OD_600nm_ of 0.2 in 50 mL of fresh YPD medium and incubated at 30°C under shaking until OD_600nm_ reached 0.7. The culture was centrifuged at 3,000 rpm for 5 min and the cell pellet was washed once in cold 10X Tris-EDTA (100 mM Tris pH 7.5, 10 mM EDTA). The cells were pelleted and resuspended in 750μL 1X LiAc buffer (100 mM lithium acetate, 10 mM Tris-HCl, 1 mM EDTA pH 7.5), then placed at room temperature for 30 minutes. One hundred microliters of competent cells were mixed with 1 to 10 μg of DNA, 50 μg of sonicated salmon sperm DNA (Sigma-Aldrich) and 700 μL of 1X LiAc supplemented with 40% polyethylene glycol 4000 (Sigma–Aldrich). The transformation mixture was then incubated overnight at 30°C. Heat-shock treatment was conducted at 44°C in a water bath for 15 minutes. Cells were gently resuspended in 3 mL YPD and incubated for 5 h to allow transformed cell recovery, prior to plating on selective media. Transformants that integrated the *caSAT1* resistance markers were selected on YPD medium supplemented with 200 μg/mL nourseothricin (Jena Bioscience) and incubated at 30°C for two days. Transformants that integrated the *hyg*B resistance marker were selected on YPD medium supplemented with 800 μg/mL hygromycin B (Sigma-Aldrich) and incubated at 30°C for two days.

### *C*. *albcians* mutant strain construction

The *C*. *albicans* mutant strains used in this study are listed in S8 Table. Oligonucleotides used for mutant creation are listed in S3 Table. To generate homozygous *ROB1* deletion mutants in clinical isolates HBJ6-2 and HBJ6-3, we used a transient CRISPR-Cas9 genome editing approach as described in Min *et al*. [30]. The Ca*CAS9* expression cassette (containing the *ENO1* promoter, Ca*CAS9* open reading frame, and *CYC1* terminator) was amplified from plasmid pV1093 [30] with primer pairs CaCas9/F and CaCas9/R (S3 Table), using DNA polymerase Ex Taq (TaKaRa, Japan). The sgRNA expression cassette containing the *SNR52* promoter, guide sequence, and sgRNA scaffold sequence was assembled by the single-joint PCR method [108]. In the first step, the *SNR52* promoter and sgRNA scaffold components were PCR amplified using flanking primers SNR52/F and sgRNA/R and internal chimeric primers SNR52/R_ROB1 and sgRNA/F_ROB1 (S3 Table). The chimeric primers overlapped by a 20-base segment that specified the guide sequence. In the second step, both components were joined by primer extension, relying upon annealing of the complementary chimeric primer extensions. In the third step, the joined product was PCR amplified with nested primers SNR52/N and sgRNA/N (S3 Table), to yield the sgRNA cassette. The repair template, carrying the *caSAT1* dominant selection marker conferring resistance to nourseothricin, was amplified from plasmid pV1093 using oligos NAT_ROB1_repair/F and NAT_ROB1_repair/R (S3 Table). Yeast cells were co-transformed with 1 to 3 μg of each of the resulting PCR-amplified DNA fragments. Correct repair template cassette integration and the deletion of both alleles of *ROB1* were confirmed by PCR, using primers flanking the expected repair template integration sites Verif-1-F, Verif-1-R, Verif-2-F, and Verif-1-R (S3 Table).

We used site-directed mutagenesis by fusion PCR to generate *ROB1*^G299E^ and *ZFU3*^A127T^ allele replacement cassettes and create *ROB1*/*ROB1*^G299E^ and *ZFU3*/*ZFU3*^A127T^ heterozygous mutants in clinical isolate HBJ6-2 (S8 Fig). The strategy relies on three fusion PCR reactions using four independently generated PCR products, namely ROB1-Frg1, ROB1-Frg2, *caSAT1*-Frg3 and Dw-Frg4 for the *ROB1*^G299E^ allele replacement cassette (S8 Fig, panels A and B); and ZFU3-Frg1, ZFU3-Frg2, *hygB*-Frg3 and Dw-Frg4 for the *ZFU3*^A127T^ allele replacement cassette (S8 Fig, panel C). All PCR reactions were performed using DNA polymerase Ex Taq (TaKaRa, Japan). For generating the *ROB1*/*ROB1*^G299E^ mutants (or respective control strains, where primers for site directed mutagenesis were not included in the design and were omitted from the PCR reactions), genomic DNA from strain HBJ6-2 and both primer pairs ROB1(I)F and ROB1(I)R (S3 Table, hybridize at positions bps 144 to 164 and bps 941 to 983 with respect to the ATG translation start site of *ROB1*, respectively) and ROB1(II)F and ROB1(II)R (S3 Table, hybridize at position bps 941 to 983 and bps 3,358 to 3,382 with resepct to the ATG translation start site of *ROB1*, respectively) were used to PCR-amplify fragments ROB1-Frg1 (840 bp, S8 Fig, panel A) and ROB1-Frg2 (2,465 bp, S8 Fig, panel A) that overlap by a 43-bp segment created by complementary primers ROB1(II)F and ROB1(I)R designed to respectively introduce G>A and C>T complementary mismatches at position 962 relative to the ATG translation start site of *ROB1* (*n*.*b*. *ROB1* carries an intron from position 48 to 113 with respect to the ATG translation start site). Fragment *caSAT1*-Frg3 (1,278 bp, S8 Fig, panel A), carrying the *caSAT1* dominant nourseothricin resistance marker for the selection of integrative transformants, was generated using pFA6-SAT1 plasmid [109] as a template and chimeric primers SAT1F (S3 Table, overlapping with primer ROB1(II)R) and SAT1R (S3 Table, overlapping with primer ROB1 downstreamF, see below). Fragment Dw-Frg4 (412 bp, S8 Fig, panel B), carrying the downstream recombination region, was PCR-amplified from strain HBJ6-2 genomic DNA using primers ROB1 downstreamF and ROB1 downstreamR (S3 Table, hybridize at positions bp 3,383 to bp 3,400 and bp 3,755 to bp 3,774 with respect to the ATG translation start site of *ROB1*, respectively). Fragments ROB1-Frg1 and ROB1-Frg2 were fused by primer extension, relying upon annealing of the complementary 43-bp segment, to yield a 3,262-bp product carrying the G>A substitution in *ROB1* and including a 340-bp sequence downstream of the TAA stop codon of *ROB1* to serve as a putative transcription termination sequence (Fusion PCR #1). Fragments *caSAT1*-Frg3 and Dw-Frg4 were fused by primer extension, relying upon annealing of the complementary 38-bp segment brought by primers ROB1 downstreamF and SAT1R, to yield a 1,650-bp product carrying the *caSAT1* selection marker and a 392-bp region allowing recombination at positions bps 341–732 downstream of the TAA stop codon of *ROB1* (Fusion PCR #2). Fusion PCR products #1 and #2 were joined in a third fusion PCR relying upon annealing of the complementary 48-bp segment brought by chimeric primer sequences ROB1(II)R and SAT1F from fragments ROB1-Frg2 and *caSAT1*-Frg3, respectively, to yield the 4,866-bp *ROB1*^G299E^ allele replacement cassette (S8 Fig, panel D). The same strategy was used to generate the 4,777-bp *ZFU3*^A127T^ allele replacement cassette (and the equivalent wild-type *ZFU3* allele replacement cassette as a control, S8 Fig, panel D). Briefly, DNA fragments ZFU3-Frg1 (738 bp, amplified with primers ZFU3(I)F and ZFU3(I)R, S3 Table), ZFU3-Frg2 (1,618 bp, amplified with primers ZFU3(II)F and ZFU3(II)R, S3 Table), *hygB*-Frg3 (2,002 bp, amplified with primers HygBF and HygBR, S3 Table) and Dw-Frg4 (568 bp, amplified with primers ZFU3 downstreamF and ZFU3 downstreamR, S3 Table) were PCR-amplified using genomic DNA from strain HBJ6-2 (for ZFU3-Frg1, ZFU3-Frg2 and Dw-Frg4) and plasmid pAU34-HygB [110] (for *hygB*-Frg3) as a template (S3 Table and S8 Fig, panel C). Fragments ZFU3-Frg1 and ZFU3-Frg2 were fused by primer extension, relying upon annealing of a complementary 46-bp segment brought by primers ZFU3(II)F and ZFU3(I)R, to yield a 2,310-bp product carrying the G>A substitution in *ZFU3* at position bp 489 downstream of the ATG translation start site and including a 620-bp sequence downstream of the TGA stop codon of *ZFU3* to serve as a putative transcription termination sequence (Fusion PCR #1). Fragments *hygB*-Frg3 and Dw-Frg4 were fused by primer extension, relying upon annealing of the complementary 49-bp segment brought by primers ZFU3-downstreamF and HygBR, to yield a 2,521-bp product carrying the *hyg*B selection marker and a 543-bp region allowing recombination at positions bps 621–1,163 downstream of the TGA stop codon of *ZFU3* (Fusion PCR #2). Fusion PCR products #1 and #2 were joined in a third fusion PCR relying upon annealing of the complementary 52-bp segment brought by chimeric primer sequences ZFU3(II)R and HygBF from ZFU3-Frg2 and *hygB*-Frg3 fragments, respectively, to yield the 4,777-bp *ZFU3*^A127T^ allele replacement cassette (S8 Fig, panel D). The correct integration of *ROB1*^G299E^ (S8 Fig, panel E) and *ZFU3*^A127^ allele (S8 Fig, panel F) replacement cassettes was confirmed with primer pairs ROB1-Veri-cassetteF and ROB1-Veri-cassetteR and ZFU3-Veri-cassetteF and ZFU3-Veri-cassetteR (S3 Table). The presence of the G>A heterozygous substitutions at positions bp 962 and bp 489 downstream of the ATG translation start sites of *ROB1* and *ZFU3* in the resulting HBJ6-2 mutants, respectively, was confirmed via Sanger sequencing (S8 Fig, panel G) using primer pairs ROB1-Veri-MutationF and ROB1-Veri-MutationR and ZFU3-Veri-MutationF and ZFU3-Veri-MutationR, respectively (S3 Table).

### Gene expression analyses by RT-PCR and qRT-PCR

The HBJ6-2 strain derivatives *ROB1*^G299E^-*SAT1*/*ROB1* and *ROB1*-*SAT1*/*ROB1* were grown overnight in liquid YPD at 30°C. The following day, cells were resuspended at an OD_600nm_ of 0.2 in liquid YPD medium and regrown for four hours under vigorous shaking at 30°C. The cultures were centrifuged at 3,000 rpm for 5 min, and the cell pellets were used for total RNA isolation with the RNeasy Mini Kit (Qiagen) according to the manufacturer’s instructions for yeast and filamentous fungi. cDNA synthesis from the extracted RNA was performed using the QuantiTect Reverse Transcription Kit (Qiagen). For each reaction, equal amounts of RNA were used (1 μg in a 20-μl reaction mixture). The expression of each gene (*ACT1*, *TEF3*, *ROB1*, *ALS3*, *HWP1* and *ECE1*) was inspected by PCR amplification (thirty cycles with 15 seconds at 95°C, 15 seconds at 50°C and 40 seconds at 70°C using Taq DNA polymerase) from the synthesized cDNA with gene-specific primers listed in S3 Table, followed by agarose gel electrophoresis of the PCR products (Fig 6A). Quantitative real-time PCR (Fig 6B) was performed in an Applied Biosystems QuantStudio 3 Real-Time PCR System, in a 25-μl reaction mixture containing 1X SYBR Green PCR Master Mix (Applied Biosystems, catalog # 4309155), 100 nM of both forward and reverse primers (see S3 Table for qPCR primers used in this study), and 1 μl of cDNA. To quantify the relative expression of the *ROB1*, *ALS3*, *HWP1*, and *ECE1* genes, the expression of endogenous gene *ACT1* was used as the normalization standard, and the relative expression of each gene in the *ROB1*^G299E^-*SAT1*/*ROB1* mutant compared to its expression in the *ROB1*-*SAT1*/*ROB1* was determined using the cycle threshold (ΔΔCt) method using the average ΔCt values of the *ACT1* gene in the *ROB1*-*SAT1*/*ROB1* strain as a calibrator. The assays were carried out three times independently, and statistical analysis was performed using a Welch’s *t*-test.

## Supporting information

S1 Fig Minimum-spanning tree profile of MLST data from *C*. *albicans* strains isolated from the airways of patients with CF. Minimum-spanning tree analysis based on MLST data from the 56 *C*. *albicans* clinical isolates from CF patients in addition to the 11 maternal isolates (67 strains in total). Each circle corresponds to a distinct allelic profile (DST), and the circle size corresponds to the number of isolates sharing the same DST. The circle was coded by assigning the same color to identical clades (Green, clade 4; blue, clade 1; yellow, clade 8; orange, clade 3; pink, clade 17 and red, clade10). The shaded zones between clusters of circles indicate that the clustered DSTs belong to the same clonal complex (*i*.*e*. clades). Numerals connecting the circles indicate the number of allelic differences between the DSTs.
(JPG)

S2 Fig Chronology of isolation of *C*. *albicans* clinical strains from the airways of patient CF02. The number of clinical isolates (*y*-axis) recovered from patient CF02 on the indicated date (*x*-axis, month abbreviation followed by year; 16, 2016; 17, 2017) during the period ranging from December 2016 to September 2017 (*x*-axis) are plotted as black histograms. The strain identifiers (starting with the letters HBJ followed by a number) are numbered according to the chronology of their sampling. When more than one isolate is recovered from a given sputum sample, an additional number separated from the strain name by a dash allows to provide a unique identifier (*e*.*g*. HBJ4 strain series were all collected in April 2017, and are composed of 5 isolates identified as HBJ4-1, HBJ4-2, HBJ4-3, HBJ4-4 and HBJ4-5). Strains isolated on a given date are listed on the corresponding histogram. For more details, see S1 Table.
(JPG)

S3 Fig Snapshots of annotated growth curves for strains HBJ6-1, HBJ6-2 and HBJ6-3 taken from TECAN Sunrise multiplate reader. Growth curves of the indicated strains (on top of each panel) generated by a TECAN Sunrise multiplate reader device display an irregular shape in stationary phase (red arrows) for strains HBJ6-1 and HBJ6-3; indicative of morphological alterations in these two isolates. The optical density at 600 nm (OD_600nm_, *y*-axis) of each culture was measured every 10 min in YPD medium at 30°C during 42 hours (*x*-axis) and was plotted as a function of time in hours (*x*-axis).
(JPG)

S4 Fig Strain HBJ6-2 forms pseudohyphae and/or short hyphae in different filamentation-inducing media. **A.** Filamentous growth of strains HBJ6-2 and HBJ6-3 (indicated on top of the panels) was assessed in RPMI (upper panel) and Spider (lower panel) liquid media, at 37°C. Thawed-out cells were incubated overnight in YPD at 30°C with shaking at 150 rpm, then harvested by centrifugation, washed once in 1X PBS and diluted to OD_600_ = 0.3 in Spider and RPMI liquid media. The cells were allowed to grow for up to 6 h at 37°C. Cells were imaged using a Leica DM500 microscope. Scale bar, 100 μm. **B.** Monospecies biofilms made by strains HBJ6-2 (middle panel) and HBJ6-3 (right panel) were allowed to grow for 24 h in DMEM medium at 37°C in polystyrene 96-well plates as described in the Materials and Methods section entitled “Dual-species biofilms of *C*. *albicans* and *P*. *aeruginosa*.”, following a protocol provided by Dr. Rebecca Hall [14,18,19]. A well devoid of *C*. *albicans* cells served as a negative control (Control). Images were captured using a Leica M80 stereomicroscope.
(JPG)

S5 Fig Density map of heterozygous single nucleotide polymorphisms (SNPs) across the genomes of 18 *C*. *albicans* isolates serially recovered from patient CF02. Each chromosome (colored horizontal bar, with chromosome number indicated on top of each bar) of the complete set of 18 *C*. *albicans* isolates serially recovered from patient CF02 was divided into 10-kb windows with colors assigned based on the number of heterozygous SNPs found between haplotypes A and B. Colors range from white (absence of heterozygous SNPs) to deep red where roughly 100 to 150 heterozygous SNPs were identified. The centromere of each chromosome is indicated with a black vertical line. The length of each chromosome is indicated by the horizontal scale bars below each chromosome depiction (kb, kilobases; Mb, megabases). The identifier of each isolate is shown on the left side of the figure. Each row represents a strain and strains are ordered according to the chronology of their isolation (*i*.*e*. oldest strain on top). The color scale of the density of heterozygous SNPs is placed on top of the figure.
(JPG)

S6 Fig Examination of chromosomal rearrangements among 18 *C*. *albicans* serial clinical isolates from patient CF02 using ABHet analysis of WGS data. Plotted are allele balance at heterozygous sites (ABHet) values (*y*-axis) ranging from 0.0 to 1.0 at each chromosomal position (*x*-axis, *C*. *albicans* chromosomes are identified on top of the figure as ChrX, where X stands for 1, 2, 3, 4, 5, 6, 7 or R) in the genomes of the 18 clinical isolates from patient CF02 (strain identifiers are indicated on left of the figure). ABHet values are defined as the number of reference reads from individuals with heterozygous genotypes divided by the total number of reads from such individuals (see Materials and Methods). A diploid genome will be defined by an ABHet value of 0.5. A triploid strain may contain either three identical alleles (an allelic frequency of 1) or two identical alleles and one different allele (frequencies of 0.66 and 0.33). A tetraploid strain may have allelic frequencies of either 0.5 (2×2 identical alleles), 1 (4 identical alleles), or 0.25 and 0.75 (3 identical alleles and 1 different allele).
(JPG)

S7 Fig Filamentous growth of strain HBJ6-3 is not inhibited in the presence of *P*. *aeruginosa* strain PAO1. *C*. *albicans strains* HBJ6-2 and HBJ6-3 were inoculated in YPD medium and *P*. *aeruginosa* strain PAO1 was inoculated in LB medium. Both were incubated at 37°C for 22 h. Overnight cultures of *C*. *albicans* and *P*. *aeruginosa* were washed twice in PBS then respectively diluted to 1×10^6^ cells/ml and to 2×10^8^ cells/ml in DMEM medium. One hundred μl of *C*. *albicans* and 10 μL of *P*. *aeruginosa* were mixed in 96-well plates and the final volume adjusted to 200 μl with DMEM. The plates were incubated for 22 h at 37°C with shaking at 150 rpm. *C*. *albicans* and *P*. *aeruginosa* co-cultures were imaged using a Leica DM500 microscope. Scale bar, 100 μm.
(JPG)

S8 Fig Strategy for the design and construction of HBJ6-2-derived *ROB1*^G299E^ and *ZFU3*^A217T^ heterozygous mutants. **A.** and **B.** Agarose gel electrophoresis of PCR amplification products used for *ROB1*^G299E^*-SAT1* cassette assembly. **A.** Lane 1, 1Kb plus DNA ladder (Thermo Scientific GeneRuler 1 kb Plus DNA Ladder); lane 2, *ROB1*-Frg1 amplicon migrates at 840 bp; lane 3, *ROB1*-Frg2 amplicon migrates at 2465 bp; lane 3, *SAT1*-Frg3 amplicon migrates at 1,278 bp. **B.** Lane 1, 1Kb plus DNA ladder (Thermo Scientific GeneRuler 1 kb Plus DNA Ladder); lane 2, Dw-Frg4 amplicon migrates at 412 bp. **C.** PCR products used for *ZFU3*^A217T^*-HygB* cassette assembly. Lane 1, 1Kb plus DNA ladder (Thermo Scientific GeneRuler 1 kb Plus DNA Ladder); lane 2, *ZFU3*-Frg1 amplicon migrates at 738 bp; lane 3, *ZFU3*-Frg2 amplicon migrates at 1,618 bp; lane 4, *hygB*-Frg3 amplicon migrates at 2,002 bp; lane 5, Dw-Frg4 amplicon migrates at 568 bp. **D.** Overlap extension PCR to construct allele replacement cassettes for *ROB1* and *ZFU3*. Lane 1, 1kb plus DNA ladder (Thermo Scientific GeneRuler 1 kb Plus DNA Ladder); lane 2, complete allele replacement cassette *ZFU3*^A217T^*-HygB* after assembling PCR products presented in panel C by fusion PCR of the four DNA fragments, with an expected size of 4,777 bp; lane3, complete allele replacement cassette *ROB1*^G299E^*-HygB* after assembling PCR products presented in panels A and B by fusion PCR of the four DNA fragments, with an expected size of 4,866 bp. **E.** The *ROB1*^G299E^*-SAT1* (upper panels) and *ZFU3*^A217T^*-HygB* (lower panels) heterozygous mutants were constructed in the HBJ6-2 strain background (upper and lower yellow depictions on the left). The *ROB1*^G299E^*-SAT1* and *ZFU3*^A217T^*-HygB* allele replacement cassettes are integrated into strain HBJ6-2 genomic DNA through recombinational exchanges (illustarted through dashed crossed lines) taking place between the *ROB1* (left upper panel) or *ZFU3* (left lower panel) alleles and the corresponding exogenous allele replacement cassettes flanked by upstream and downstream (DW) homology regions. Markers conferring resistance to nourseothricin (upper panel, *caSAT1*, purple rectangle) and hygromycin B (lower panel, *caHygB*, blue rectangle) are illustrated flanked by their respective promoter and terminator sequences (P and T, respectively). Growth of nourseothricin- (upper middle panel, *ROB1*/*ROB1*^G299E^) and hygromicin B- (lower middle panel, *ZFU3*/*ZFU3*^A217T^) resistant transformants on selective media is followed by colony PCR analysis to screen for heterozygous mutants (right panels). Positive *ROB1*/*ROB1*^G299E^ clones C1, C4 and C5 (upper right panel, yellow stars) display a PCR-amplified fragment of 1,003 bp, whereas positive *ZFU3*/*ZFU3*^A217T^ clones C1-C5 (lower right panel, yellow stars) display a PCR-amplified fragment of 594. **G.** Heterozygous single nucleotide substitutions in *ROB1* and *ZFU3* were confirmed via Sanger sequencing (yellow stars) The heterozygous mutations are indicated as double peaks at the same position within the sequencing chromatograms. Forward sequencing (F, upper panels) and reverse sequencing (R, lower panels) show similar results, validating the generation of *ROB1*/*ROB1*^G299E^ and *ZFU3*/*ZFU3*^A217T^ HBJ6-2 mutant derivatives.
(JPG)

S1 Table List of patients recruited in this study. **Patient ID**, a specific number was assigned to each patient according to the chronology of their recruitment; **Age**, patient age in years; **Sex**, patient sex (M, male; F, female); **Sampling date**, date/month/year on which sampling was performed; **Sample #**, sample number according to the chronology of sampling; **Sample from**, body area from which sample was obtained (Exp. Sputum, expectorated sputum); ***Candida* culture**, indicates if culture was performed and whether yeast colonies were obtained. *Candida*, isolation of *Candida* spp. using a culture-based method at the Division of Mycology, La Rabta Hospital Tunis, relying on colony growth on Sabouraud’s medium supplemented with chloramphenicol and actidione, followed by chlamydosporulation testing (see Materials and Methods); **# of *Candida* isolates**, total number of *Candida* isolates from each sputum/oral/vaginal sample; **Species ID**, *Candida* species identified using MALDI ToF or ITS sequencing approaches or a combination of both; **Method**, method used for species identification; ***C*. *albicans* strain ID**, strain nomenclature according to the chronology of sampling date; **DST, clade**, Diploid sequence type of the isolate based on MLST data, followed by the clade to which the isolate belongs; ***Aspergillus***, indicates if an *Aspergillus* species was co-isolated with *Candida* species from the same sample; ***Pseudomonas***, indicates if *Pseudomonas aeruginosa* was co-isolated with *Candida* species from the same sample; **Other bacteria**, indicates if other bacteria species were co-isolated with *Candida* species from the same sample; **N/A**, not applicable; **ND**, not determined.
(XLSX)

S2 Table Clinical parameters of CF patients recruited in this study. **Patient ID**, a specific number was assigned to each patient according to the chronology of their recruitment; **Mutation**, CFTR mutation for each patient was provided by the group of Pr. Taieb Messaoud at Children’s Hospital in Tunis. Confirmed CF diagnosis was established based on clinical symptoms with two positive sweat chloride tests, sequencing of the CFTR gene and identification of the CF-causing mutation; **BMI**, body mass index for each patient, defined as the body weight in kilograms divided by the square of height in meters; ***P*. *aeruginosa***, **colonization**, chronicity of *P*. *aeruginosa* carriage (chronic, intermittent or undetectable); **Mucoid *P*. *aeruginosa***, presence or absence of mucoid form of *P*. *aeruginosa* (Yes, presence; No, absence); **ABPA**, presence (Yes) or absence (No) of allergic bronchopulmonary aspergillosis; **Bronchiectasis**, absence (No) or presence of bronchiectasis in one, two (1 or 2 lobes) or more (≥ 2 lobes) lobes; ***Burkholderia cepacia***, detection (Yes) or undetectable (No) of *Burkholderia cepacia* infection; **Respiratory exacerbation**, number of times patient was admitted in ICU for severe respiratory exacerbation; **Cirrhosis**, absence (No) or presence (Yes) of hepatic cirrhosis; **Severity score**, a severity score was calculated based on arbitrary scoring scheme provided in bottom table.
(XLSX)

S3 Table Primers used in this study. Includes 6 sheets listing primers used for MLST analyses (sheet #1, named MLST primers), ITS2 sequencing for species identification (sheet #2, named ITS2 primers), the generation of the *ROB1*/*ROB1*^G299E^ heterozygous mutants in strain HBJ6-2 (sheet #3, named *ROB1* primers), the generation of the *ZFU3*/*ZFU3*^A217T^ heterozygous mutants in strain HBJ6-2 (sheet #4, named *ZFU3* primers), the generation of *rob1*Δ/*rob1*Δ homozygous mutants in HBJ62 and HBJ6-3 isolates (sheet #5, named *ROB1* deletion primers) and RT-PCR and qRT-PCR analyses (sheet #6, named (q)RT-PCR primers).
(XLSX)

S4 Table Complete list of single nucleotide polymorphisms across 18 isolates from patient CF02. **Chr-Position**, position of SNPs on the indicated chromosome (Ca22chrx, where x designates chromosome number, followed by position of SNP on the corresponding chromosome) according to the *C*. *albicans* genome Assembly 22, version A22-s07-m01-r57; **SC5314**, polymorphic nucleotide in haplotypes A and B of the reference genome; **HBJ1 to HBJ8-4**, SNPs (haplotypes A and B) relative to polymorphic nucleotide from strain SC5314 are listed for each of the 18 serially recovered clinical isolates from patient CF02.
(XLSX)

S5 Table Pairwise comparison of the number of SNPs among the 18 serial isolates from patient CF02. A matrix listing the set of 18 serial isolates from patient CF02 lists the number of SNPs differentiating each isolate from the other. The average number of SNPs for each isolate when compared to each of the remaining ones is shown on the bottom of each column.
(XLSX)

S6 Table List of 239 single nucleotide polymorphisms exclusively shared between strains HBJ4-3, HBJ6-1 and HBJ6-3. **Chr-Position**, position of SNPs on the indicated chromosome (Ca22chrx, where x designates chromosome number, followed by position of SNP on the corresponding chromosome) according to the *C*. *albicans* genome Assembly 22, version A22-s07-m01-r57; **ORF name**, systematic nomenclature of ORF, including intron or exon region, where are located differential SNPs (haplotypes A and B) in strains HBJ4-3, HBJ6-1 and HBJ6-3 relative to those present in the remaining 15 CF02 isolates; **Gene name**, name of the gene corresponding to ORF/intron/exon where the SNPs are located; **Location of SNP**, location of SNP in the genome, either in intergenic/repeat or ORF/exon/intron regions; **Polymorphism**, nature of differential polymorphism in strains HBJ4-3, HBJ6-1 and HBJ6-3 relative to those present in the remaining 15 CF02 isolates; **SC5314**, polymorphic nucleotide in haplotypes A and B of the reference genome; **HBJ1 to HBJ8-4**, SNPs (haplotypes A and B) relative to polymorphic nucleotide from strain SC5314 are listed for each of the 18 serially recovered clinical isolates from patient CF02.
(XLSX)

S7 Table List of 34 non-synonymous single nucleotide polymorphisms exclusively shared between strains HBJ4-3, HBJ6-1 and HBJ6-3. **Chr-Position**, position of non-synonymous SNPs on the indicated chromosome (Ca22chrx, where x designates chromosome number, followed by position of SNP on the corresponding chromosome) according to the *C*. *albicans* genome Assembly 22, version A22-s07-m01-r57; **ORF name**, systematic nomenclature of ORF where are located differential non-synonymous SNPs (haplotypes A and B) in strains HBJ4-3, HBJ6-1 and HBJ6-3 relative to SNPs present in the remaining 15 CF02 isolates; **Gene name**, name of the gene corresponding to ORF where the non-synonymous SNPs are located; **Polymorphism**, nature of differential polymorphism in strains HBJ4-3, HBJ6-1 and HBJ6-3 relative to those present in the remaining 15 CF02 isolates; **Amino acid change**, deduced amino acid change in the predicted protein sequence of the ORF where the non-synonymous SNP from strains HBJ4-3, HBJ6-1 and HBJ6-3 is located; **SC5314**, polymorphic nucleotide in haplotypes A and B of the reference genome; **HBJ1 to HBJ8-4**, SNPs (haplotypes A and B) relative to polymorphic nucleotide from strain SC5314 are listed for each of the 18 serially recovered clinical isolates from patient CF02. The non-synonymous SNPs carried by strains HBJ4-3, HBJ6-1 and HBJ6-3 are highlighted in light gray.
(XLSX)

S8 Table List of *C*. *albicans* parental and mutant strains used in this study. This table lists all parental and mutant strains used in this study, including detailed information on genotypes (column #4, relevant genotype) as well as the clone number (column #2) for each independent mutant strain generated.
(XLSX)
