# Supplementary figures and images for: A gain-of-function mutation in zinc cluster transcription factor Rob1 drives Candida albicans adaptive growth in the cystic fibrosis lung environment
Source: PLoS Pathog. 2024 Apr 11;20(4):e1012154. doi: 10.1371/journal.ppat.1012154 (PMC11037546; doi:10.1371/journal.ppat.1012154)

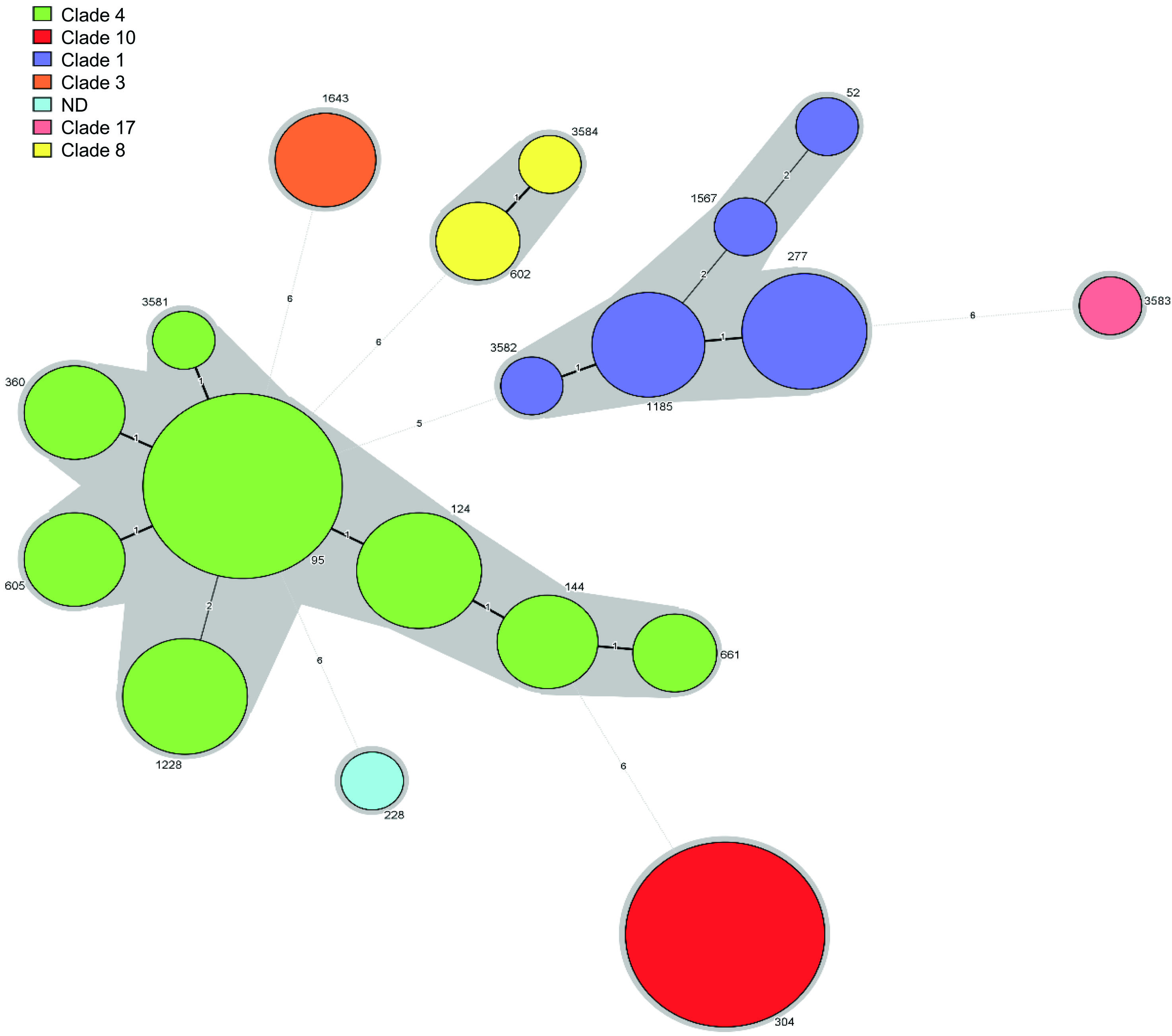

Supplement: S1 Fig — Minimum-spanning tree analysis based on MLST data from the 56 C. albicans clinical isolates from CF patients in addition to the 11 maternal isolates (67 strains in total). Each circle corresponds to a distinct allelic profile (DST), and the circle size corresponds to the number of isolates sharing the same DST. The circle was coded by assigning the same color to identical clades (Green, clade 4; blue, clade 1; yellow, clade 8; orange, clade 3; pink, clade 17 and red, clade10). The shaded zones between clusters of circles indicate that the clustered DSTs belong to the same clonal complex (i.e. clades). Numerals connecting the circles indicate the number of allelic differences between the DSTs. (JPG) [file ppat.1012154.s001.jpg]

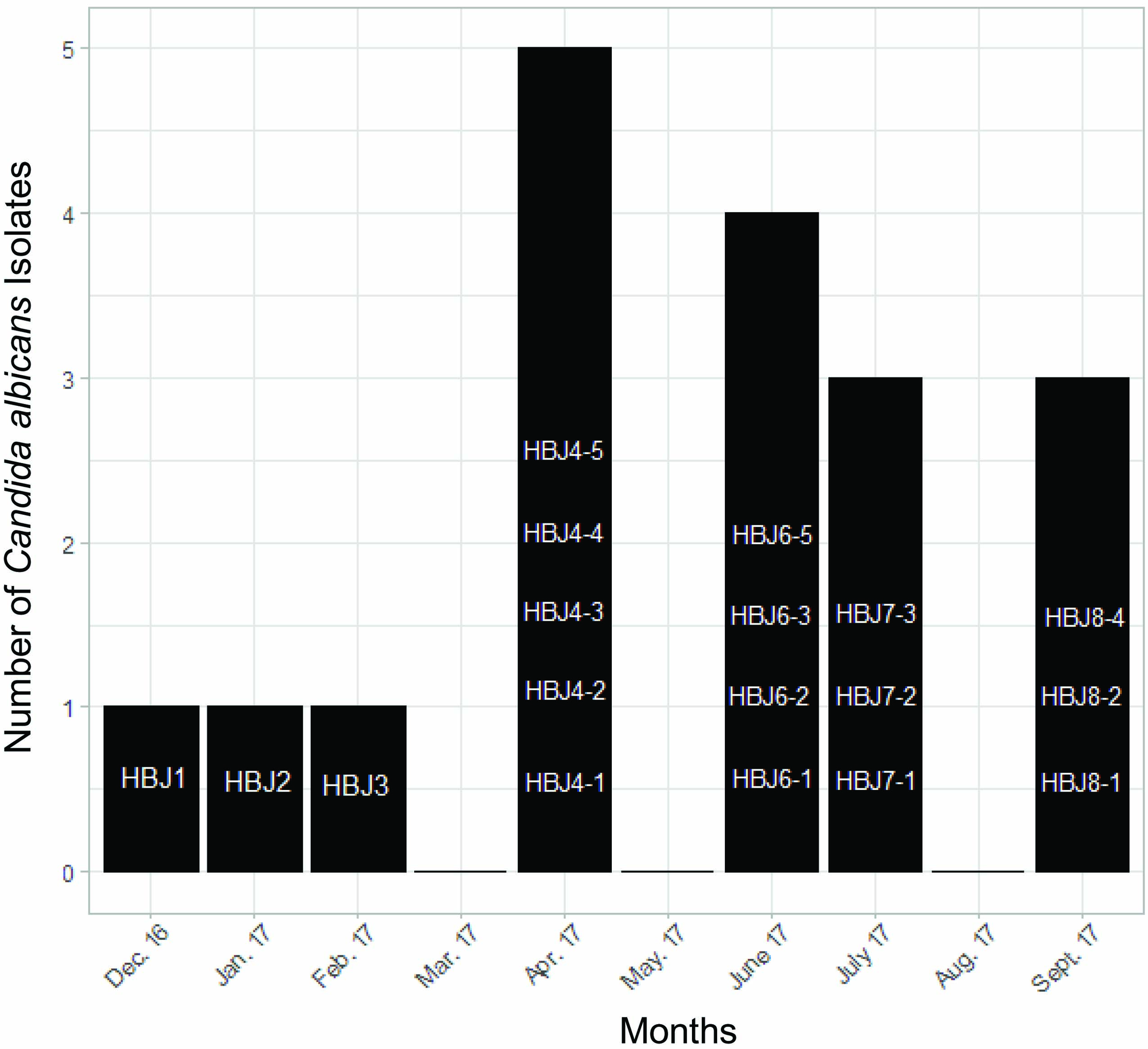

Supplement: S2 Fig — The number of clinical isolates (y-axis) recovered from patient CF02 on the indicated date (x-axis, month abbreviation followed by year; 16, 2016; 17, 2017) during the period ranging from December 2016 to September 2017 (x-axis) are plotted as black histograms. The strain identifiers (starting with the letters HBJ followed by a number) are numbered according to the chronology of their sampling. When more than one isolate is recovered from a given sputum sample, an additional number separated from the strain name by a dash allows to provide a unique identifier (e.g. HBJ4 strain series were all collected in April 2017, and are composed of 5 isolates identified as HBJ4-1, HBJ4-2, HBJ4-3, HBJ4-4 and HBJ4-5). Strains isolated on a given date are listed on the corresponding histogram. For more details, see S1 Table. (JPG) [file ppat.1012154.s002.jpg]

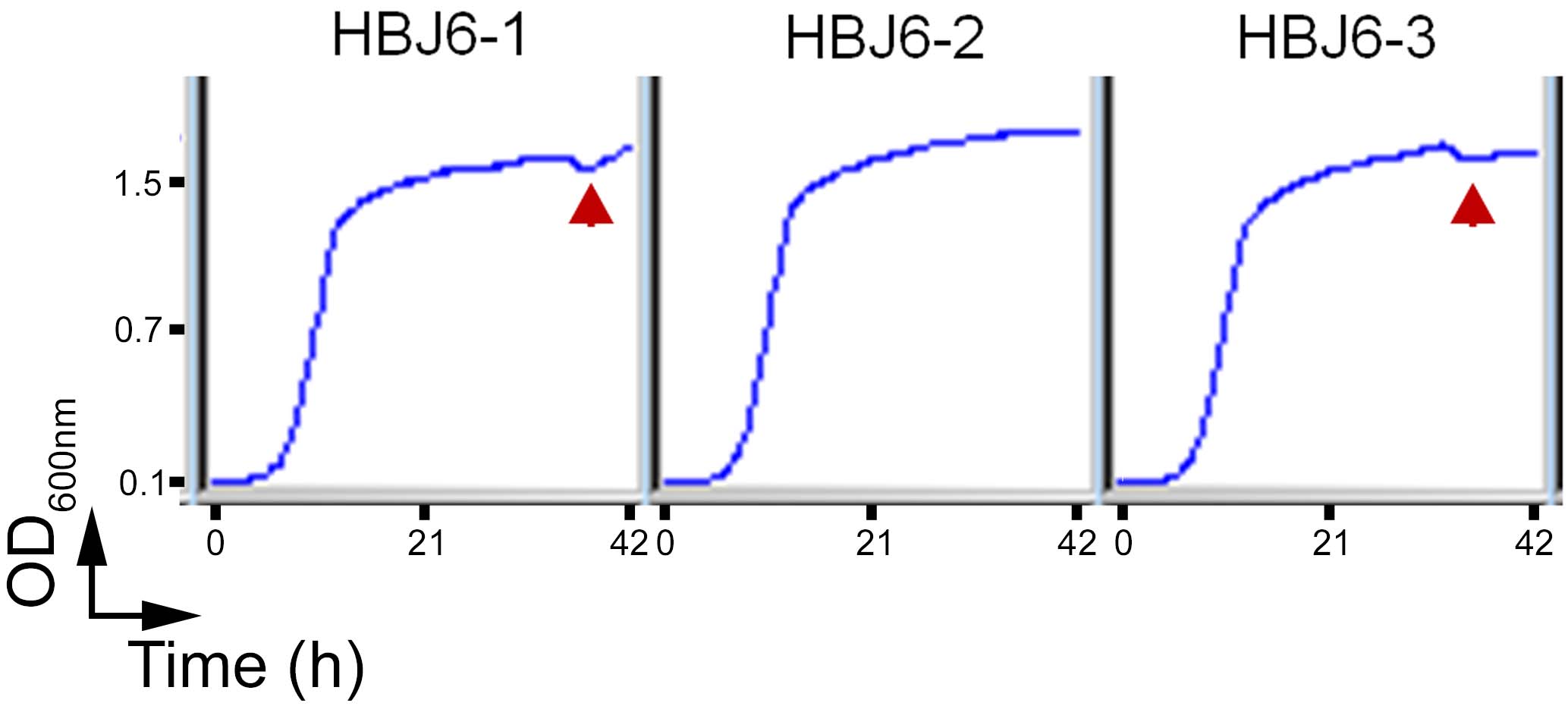

Supplement: S3 Fig — Growth curves of the indicated strains (on top of each panel) generated by a TECAN Sunrise multiplate reader device display an irregular shape in stationary phase (red arrows) for strains HBJ6-1 and HBJ6-3; indicative of morphological alterations in these two isolates. The optical density at 600 nm (OD600nm, y-axis) of each culture was measured every 10 min in YPD medium at 30°C during 42 hours (x-axis) and was plotted as a function of time in hours (x-axis). (JPG) [file ppat.1012154.s003.jpg]

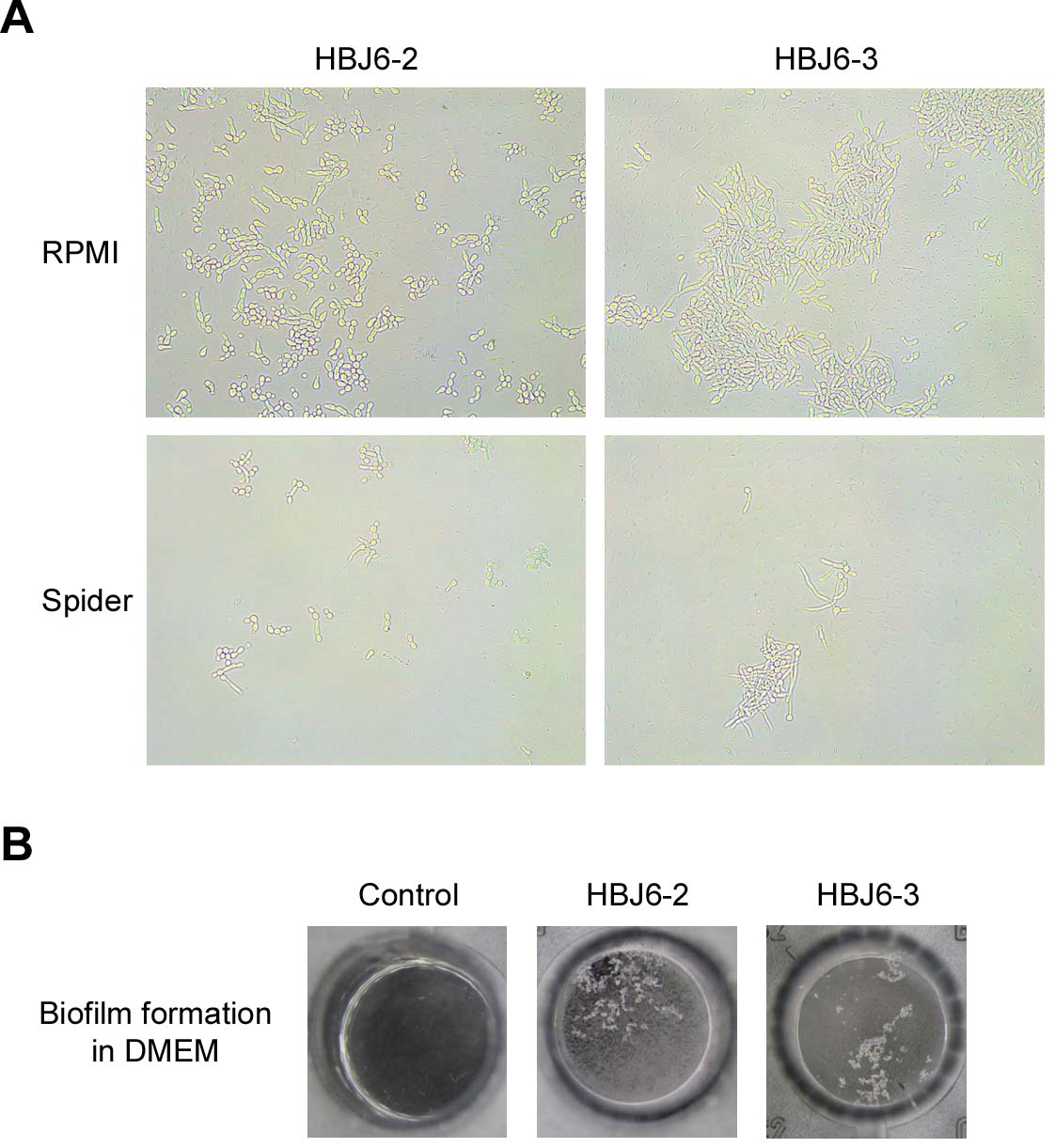

Supplement: S4 Fig — A. Filamentous growth of strains HBJ6-2 and HBJ6-3 (indicated on top of the panels) was assessed in RPMI (upper panel) and Spider (lower panel) liquid media, at 37°C. Thawed-out cells were incubated overnight in YPD at 30°C with shaking at 150 rpm, then harvested by centrifugation, washed once in 1X PBS and diluted to OD600 = 0.3 in Spider and RPMI liquid media. The cells were allowed to grow for up to 6 h at 37°C. Cells were imaged using a Leica DM500 microscope. Scale bar, 100 μm. B. Monospecies biofilms made by strains HBJ6-2 (middle panel) and HBJ6-3 (right panel) were allowed to grow for 24 h in DMEM medium at 37°C in polystyrene 96-well plates as described in the Materials and Methods section entitled “Dual-species biofilms of C. albicans and P. aeruginosa.”, following a protocol provided by Dr. Rebecca Hall [14,18,19]. A well devoid of C. albicans cells served as a negative control (Control). Images were captured using a Leica M80 stereomicroscope. (JPG) [file ppat.1012154.s004.jpg]

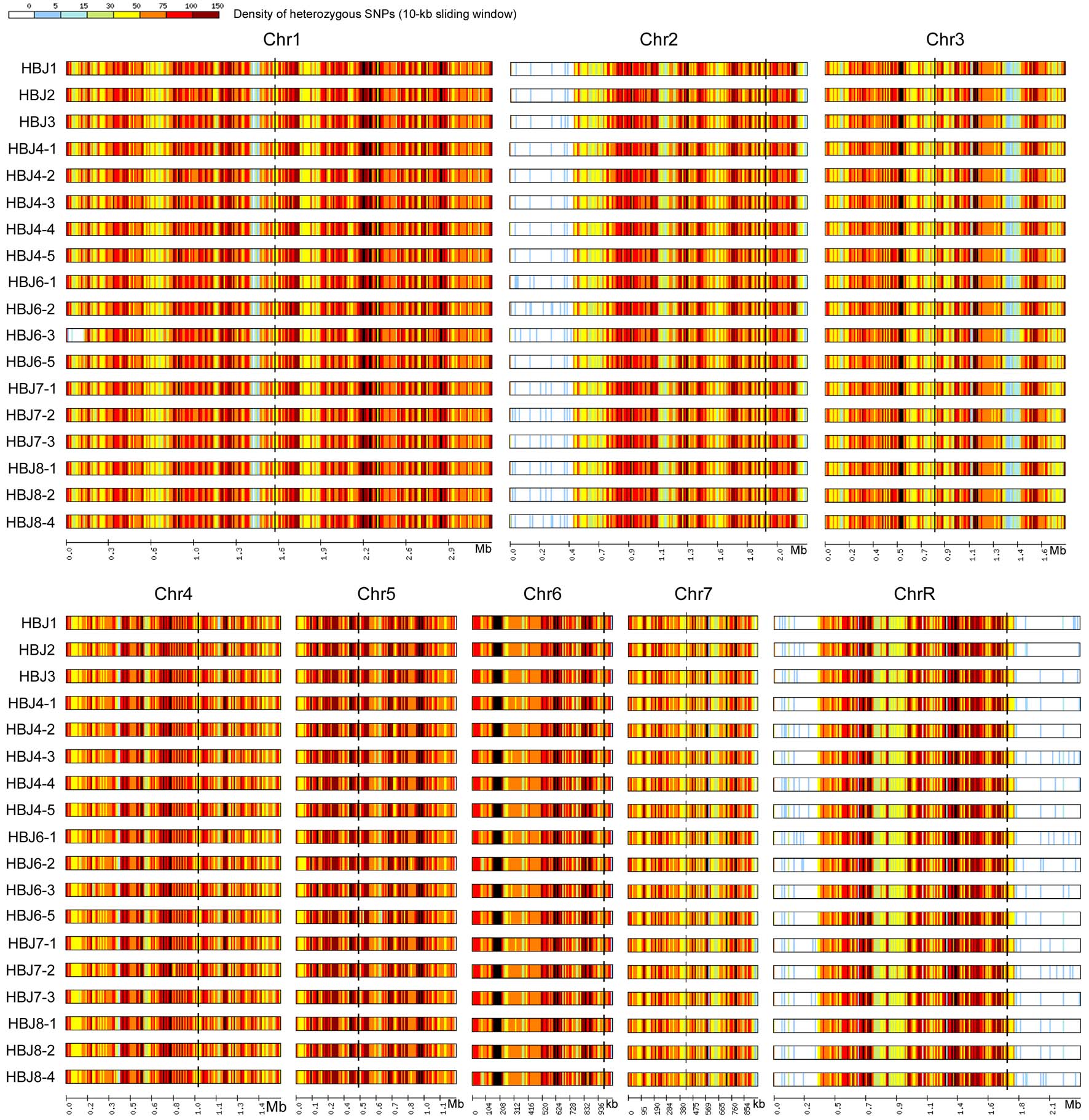

Supplement: S5 Fig — Each chromosome (colored horizontal bar, with chromosome number indicated on top of each bar) of the complete set of 18 C. albicans isolates serially recovered from patient CF02 was divided into 10-kb windows with colors assigned based on the number of heterozygous SNPs found between haplotypes A and B. Colors range from white (absence of heterozygous SNPs) to deep red where roughly 100 to 150 heterozygous SNPs were identified. The centromere of each chromosome is indicated with a black vertical line. The length of each chromosome is indicated by the horizontal scale bars below each chromosome depiction (kb, kilobases; Mb, megabases). The identifier of each isolate is shown on the left side of the figure. Each row represents a strain and strains are ordered according to the chronology of their isolation (i.e. oldest strain on top). The color scale of the density of heterozygous SNPs is placed on top of the figure. (JPG) [file ppat.1012154.s005.jpg]

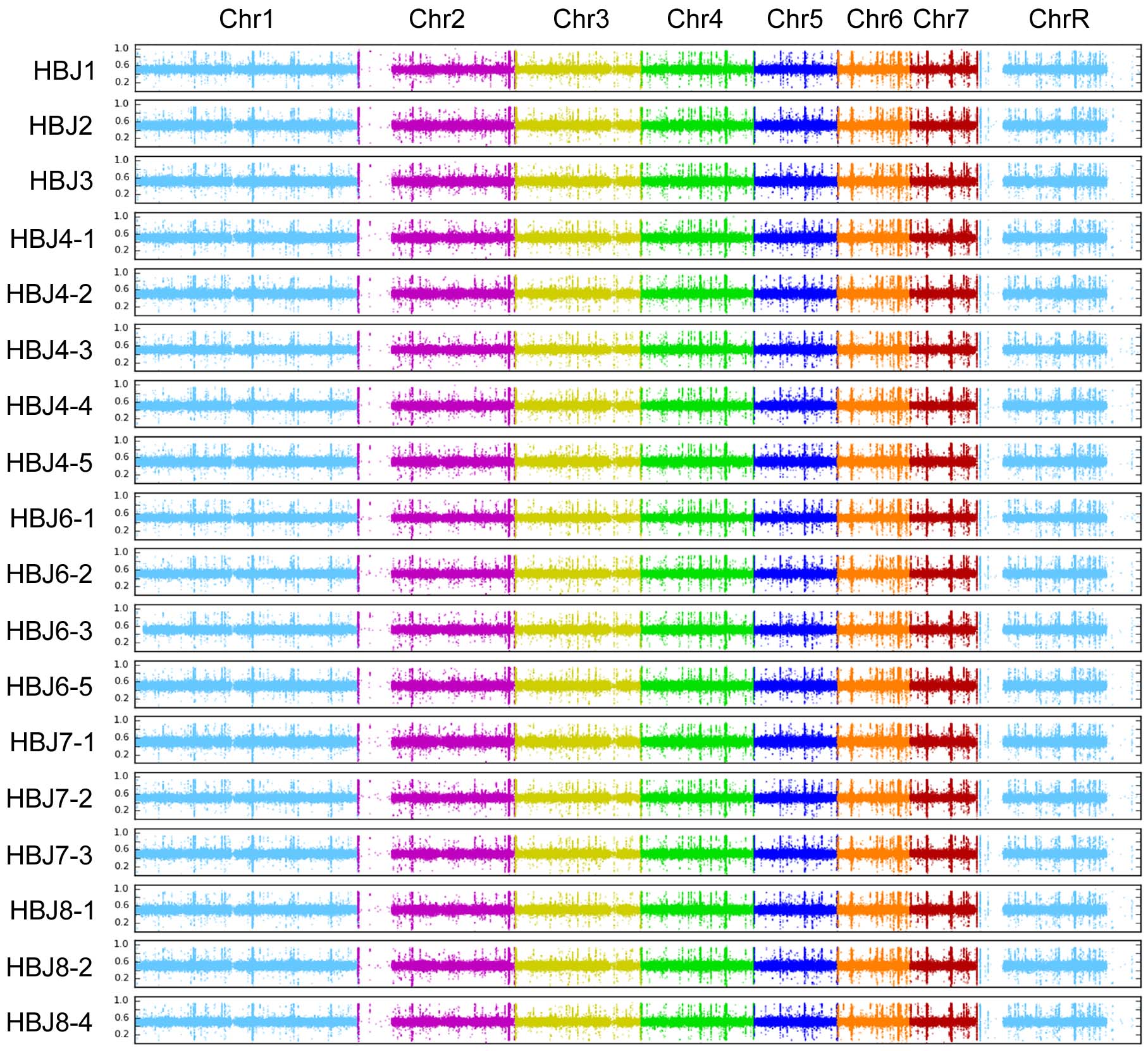

Supplement: S6 Fig — Plotted are allele balance at heterozygous sites (ABHet) values (y-axis) ranging from 0.0 to 1.0 at each chromosomal position (x-axis, C. albicans chromosomes are identified on top of the figure as ChrX, where X stands for 1, 2, 3, 4, 5, 6, 7 or R) in the genomes of the 18 clinical isolates from patient CF02 (strain identifiers are indicated on left of the figure). ABHet values are defined as the number of reference reads from individuals with heterozygous genotypes divided by the total number of reads from such individuals (see Materials and Methods). A diploid genome will be defined by an ABHet value of 0.5. A triploid strain may contain either three identical alleles (an allelic frequency of 1) or two identical alleles and one different allele (frequencies of 0.66 and 0.33). A tetraploid strain may have allelic frequencies of either 0.5 (2×2 identical alleles), 1 (4 identical alleles), or 0.25 and 0.75 (3 identical alleles and 1 different allele). (JPG) [file ppat.1012154.s006.jpg]

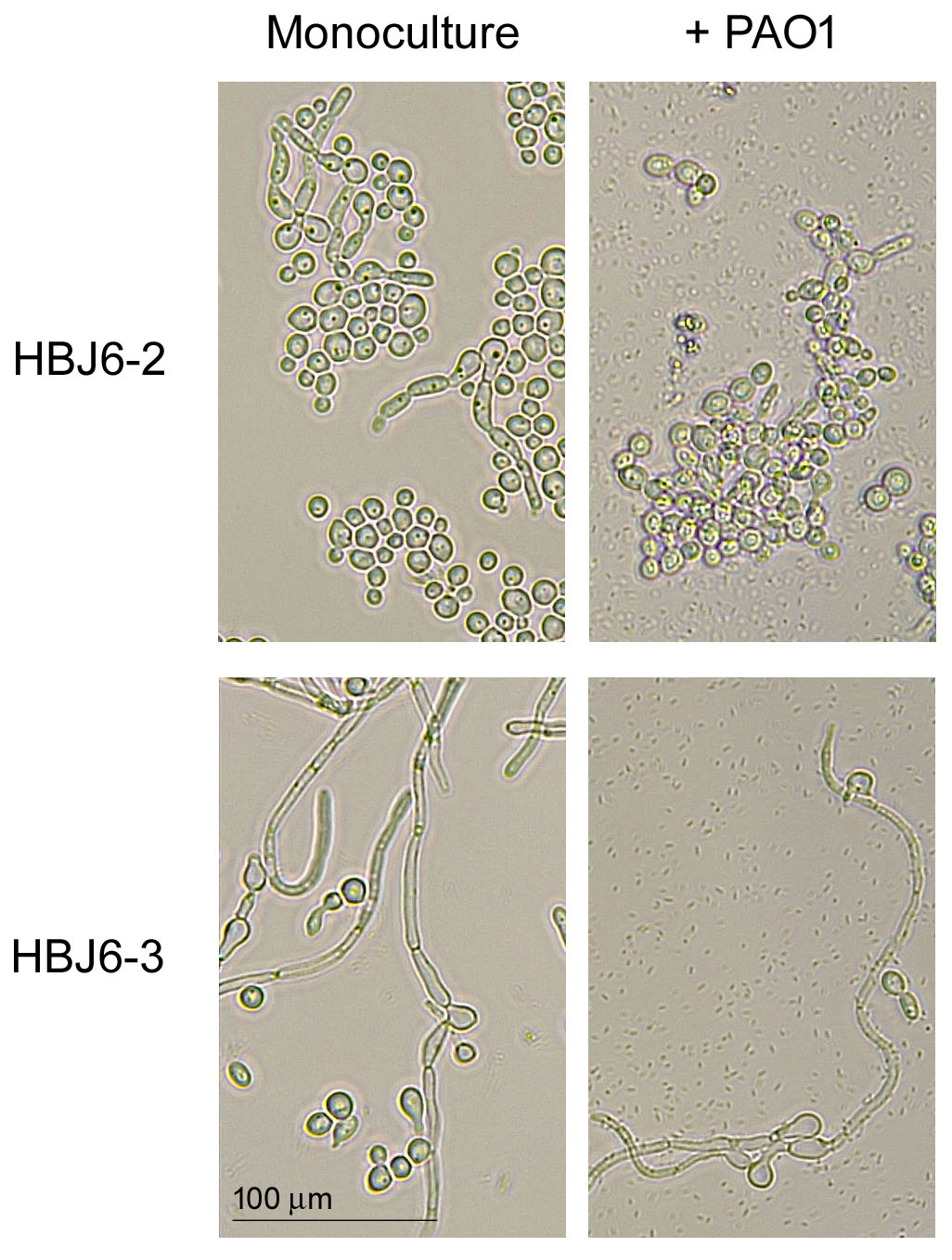

Supplement: S7 Fig — C. albicans strains HBJ6-2 and HBJ6-3 were inoculated in YPD medium and P. aeruginosa strain PAO1 was inoculated in LB medium. Both were incubated at 37°C for 22 h. Overnight cultures of C. albicans and P. aeruginosa were washed twice in PBS then respectively diluted to 1×10^6^ cells/ml and to 2×10^8^ cells/ml in DMEM medium. One hundred μl of C. albicans and 10 μL of P. aeruginosa were mixed in 96-well plates and the final volume adjusted to 200 μl with DMEM. The plates were incubated for 22 h at 37°C with shaking at 150 rpm. C. albicans and P. aeruginosa co-cultures were imaged using a Leica DM500 microscope. Scale bar, 100 μm. (JPG) [file ppat.1012154.s007.jpg]

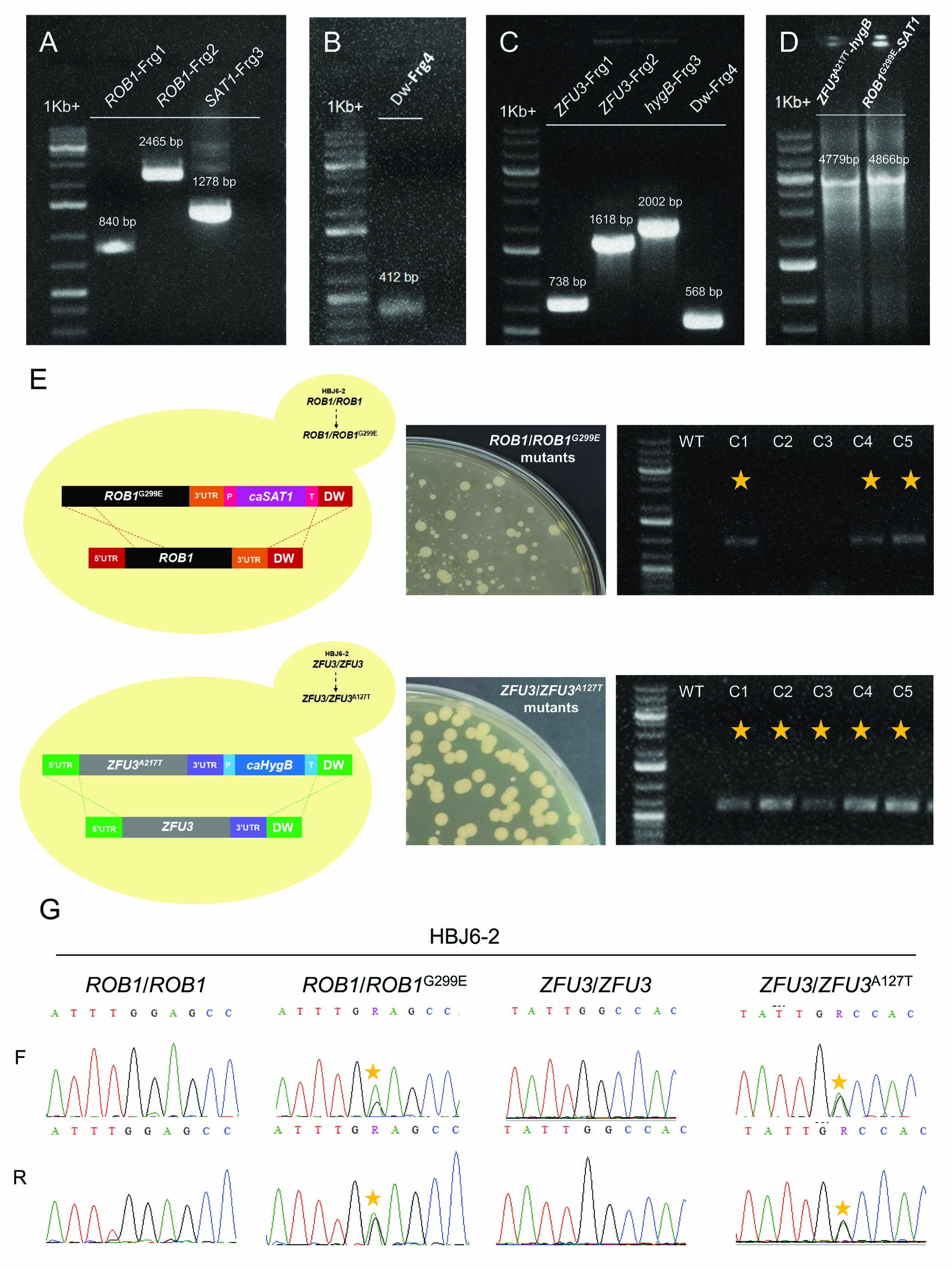

Supplement: S8 Fig — A. and B. Agarose gel electrophoresis of PCR amplification products used for ROB1G299E-SAT1 cassette assembly. A. Lane 1, 1Kb plus DNA ladder (Thermo Scientific GeneRuler 1 kb Plus DNA Ladder); lane 2, ROB1-Frg1 amplicon migrates at 840 bp; lane 3, ROB1-Frg2 amplicon migrates at 2465 bp; lane 3, SAT1-Frg3 amplicon migrates at 1,278 bp. B. Lane 1, 1Kb plus DNA ladder (Thermo Scientific GeneRuler 1 kb Plus DNA Ladder); lane 2, Dw-Frg4 amplicon migrates at 412 bp. C. PCR products used for ZFU3A217T-HygB cassette assembly. Lane 1, 1Kb plus DNA ladder (Thermo Scientific GeneRuler 1 kb Plus DNA Ladder); lane 2, ZFU3-Frg1 amplicon migrates at 738 bp; lane 3, ZFU3-Frg2 amplicon migrates at 1,618 bp; lane 4, hygB-Frg3 amplicon migrates at 2,002 bp; lane 5, Dw-Frg4 amplicon migrates at 568 bp. D. Overlap extension PCR to construct allele replacement cassettes for ROB1 and ZFU3. Lane 1, 1kb plus DNA ladder (Thermo Scientific GeneRuler 1 kb Plus DNA Ladder); lane 2, complete allele replacement cassette ZFU3A217T-HygB after assembling PCR products presented in panel C by fusion PCR of the four DNA fragments, with an expected size of 4,777 bp; lane3, complete allele replacement cassette ROB1G299E-HygB after assembling PCR products presented in panels A and B by fusion PCR of the four DNA fragments, with an expected size of 4,866 bp. E. The ROB1G299E-SAT1 (upper panels) and ZFU3A217T-HygB (lower panels) heterozygous mutants were constructed in the HBJ6-2 strain background (upper and lower yellow depictions on the left). The ROB1G299E-SAT1 and ZFU3A217T-HygB allele replacement cassettes are integrated into strain HBJ6-2 genomic DNA through recombinational exchanges (illustarted through dashed crossed lines) taking place between the ROB1 (left upper panel) or ZFU3 (left lower panel) alleles and the corresponding exogenous allele replacement cassettes flanked by upstream and downstream (DW) homology regions. Markers conferring resistance to nourseothricin (upper panel, caSAT1, purple [file ppat.1012154.s008.jpg]
